# A High-Throughput Toxicity Screen of 42 Per- and Polyfluoroalkyl Substances (PFAS) and Functional Assessment of Migration and Gene Expression in Human Placental Trophoblast Cells

**DOI:** 10.3389/ftox.2022.881347

**Published:** 2022-04-25

**Authors:** Bevin E. Blake, Brittany P. Rickard, Suzanne E. Fenton

**Affiliations:** ^1^ Curriculum in Toxicology and Environmental Medicine, University of North Carolina at Chapel Hill, Chapel Hill, NC, United States; ^2^ Mechanistic Toxicology Branch, Division of the National Toxicology Program, National Institute of Environmental Health Sciences, Research Triangle Park, NC, United States

**Keywords:** *In Vitro* toxicity, trophoblasts, PFAS, high-throughput (HT) testing, alternative methods

## Abstract

Per- and polyfluoroalkyl substances (PFAS) have become ubiquitous environmental contaminants that have been associated with adverse pregnancy outcomes in women and experimental research models. Adverse developmental and reproductive outcomes have been investigated for relatively few PFAS, and such studies are not scalable to address the thousands of unique chemical structures. As the placenta has been reported as a PFAS target tissue, the human placental trophoblast JEG-3 cell line was employed in a high-throughput toxicity screen (HTTS) to evaluate the effects of 42 unique PFAS on viability, proliferation, and mitochondrial membrane potential (MMP). HTTS concentration-response curve fitting determined EC50 values for 79% of tested compounds for at least one of the three endpoints. Trophoblast migratory potential was evaluated for a subset of six prioritized PFAS using a scratch wound assay. Migration, measured as the percent of wound closure after 72 h, was most severely inhibited by exposure to 100 µM perfluorooctanoic acid (PFOA; 72% closure), perfluorooctanesulfonic acid (PFOS; 57% closure), or ammonium perfluoro-2-methyl-3-oxahexanoate (GenX; 79% closure). PFOA and GenX were subsequently evaluated for disrupted expression of 46 genes reported to be vital to trophoblast health. Disrupted regulation of oxidative stress was suggested by altered expression of *GPEX1* (300 µM GenX and 3 µM GenX), *GPER1* (300 µM GenX), and *SOD1* and altered cellular response to xenobiotic stress was indicated by upregulation of the placental efflux transporter, *ABCG2* (300 µM GenX, 3 µM GenX, and 100 µM PFOA). These findings suggest the placenta is potentially a direct target of PFAS exposure and indicate that trophoblast cell gene expression and function are disrupted at PFAS levels well below the calculated cytotoxicity threshold (EC50). Future work is needed to determine the mechanism(s) of action of PFAS towards placental trophoblasts.

## Introduction

Per- and polyfluoroalkyl substances (PFAS) are a large family of “fluorinated substances that contain at least one fully fluorinated methyl or methylene carbon atom” (Wang et al., 2021), and they comprise over 10,000 unique chemical structures (EPA CompTox Database, accessed on 8 March 2022; Williams et al., 2017). Some PFAS have become ubiquitous in the environment (Kaboré et al., 2018; Pan et al., 2018), detectable in biological matrices across humans and wildlife around the globe (Houde et al., 2006), and contaminants in air, food, and drinking water (Sunderland et al., 2019; De Silva et al., 2021; Guelfo et al., 2021).

The physiological demands of pregnancy require a higher daily intake of water relative to non-pregnant adults (Zhang et al., 2020), putting them and their developing offspring at greater risk for exposure to drinking water contaminants (Post et al., 2017; Fenton et al., 2021; Rickard et al., 2022). Pregnant women and their developing offspring are thought to be particularly vulnerable to PFAS due to associations between maternal PFAS exposure and adverse pregnancy outcomes, such as low birth weight (Fei et al., 2007; Chen et al., 2012; Johnson et al., 2014), increased gestational weight gain (Ashley-Martin et al., 2016), preeclampsia (Huang et al., 2019; Wikström et al., 2019; Bommarito et al., 2021), gestational hypertension (Holtcamp, 2012; Huang et al., 2019), and gestational diabetes (Matilla-Santander et al., 2017; Wang et al., 2018). The placenta is involved in the etiology of these pregnancy conditions (Pijnenborg et al., 1991) and PFAS pass from the maternal to fetal compartments through the placenta (Chen et al., 2017; Zhao et al., 2017; Wang et al., 2019). Perfluorooctanoic acid (PFOA) and ammonium perfluoro-2-methyl-3-oxahexanoate (the ammonium carboxylate salt of the chemical compound hexafluoropropylene oxide-dimer acid, HFPO-DA, commonly referred to as GenX) have been shown to cause histopathological lesions in the mouse placenta (Blake et al., 2020; Jiang et al., 2020). While it is not well understood how maternal exposure to a few PFAS may contribute to adverse pregnancy outcomes, their effect(s) on placental function is likely critical and may extend to other untested PFAS (Blake and Fenton, 2020; Rickard et al., 2022).

Proper development of the placenta is essential for the establishment of maternal:fetal nutrient/waste transfer and maintenance of pregnancy and disruptions in its development or function, such as placental insufficiency, are detrimental to maternal-fetal health (Regnault et al., 2002; Gagnon, 2003; Chaddha et al., 2004). Placental insufficiency broadly describes pregnancy complications of placental origin that occur when the placenta is unable to deliver an adequate supply of nutrients and oxygen to the fetus (Gagnon, 2003; Chaddha et al., 2004). Environmental pollutants have been previously shown to increase risk for placenta-mediated pregnancy complications (Laine et al., 2015; National Toxicology Program, 2019; Philippat et al., 2019), including maternal exposure to certain PFAS, such as PFOA (Stein et al., 2009), perfluorooctane sulfonate (PFOS) (Wikström et al., 2019), and perfluorobutanesulfonate (PFBS) (Huang et al., 2019). However, the contributors to and initiating events of placental toxicity of even well-studied PFAS like PFOA and PFOS is not well understood. There is an immense knowledge gap regarding the placental toxicity of the more than 600 PFAS compounds currently in United States commerce (Blake and Fenton, 2020; Environmental Protection Agency, 2020). There is also an urgent need for data on emerging and alternative chemistry PFAS such as HFPO-DA (known as GenX), among others, which have impacted highly-exposed communities (Sun et al., 2016; Gebbink and van Leeuwen, 2020; Kotlarz et al., 2020). The rapidly expanding universe of PFAS, as well as the critical need for data on emerging replacements to the legacy PFAS, requires the use of *in silico* or *in vitro* screening models to prioritize those PFAS most urgently needing animal studies to protect public health.

*In vitro* experimental systems are an affordable and efficient way to approach complicated multi-compound toxicity screening efforts. Human-derived JEG-3 placental trophoblasts have been previously implemented in toxicity studies of environmental exposures representing a model system of the placenta (Huang and Leung, 2009; Gorrochategui et al., 2014; Adebambo et al., 2018; Bangma et al., 2020a; Olivier et al., 2021), including their use in migration assays (Brooks and Fry, 2017). The JEG-3 cell line is particularly relevant as it is composed of multinucleated trophoblasts, which facilitate the exchange of nutrients, waste, and gases between the maternal and fetal compartments in human placenta. The mechanism(s) through which PFAS alter fetal growth via disruption of placental function is not well understood but may occur due to alterations in placental gene expression pathways involved in nutrient or xenobiotic transport (Birru et al., 2021), oxidative stress (Du et al., 2021), endocrine function (Suh et al., 2011), and metabolism (Szilagyi et al., 2020). There are limited data on the ability of PFAS to induce gene expression changes in the placenta, but previous work has shown JEG-3 exposure to PFOA or PFOS inhibits aromatase activity *in vitro* (Gorrochategui et al., 2014) and PFOA exposure during gestation *in vivo* decreases expression of placental prolactin-family genes in mice (Suh et al., 2011).

This study leveraged JEG-3 placental trophoblast cells to execute a multiplexed high-throughput toxicity screen to evaluate a panel of 42 PFAS. The aim was to generate robust concentration-response data corresponding to cell viability, proliferation, and mitochondrial membrane potential, and extract relevant model parameters (e.g., EC50 values) to further characterize and categorize PFAS compounds by bioactivity and chemical features. Two compounds of high public health interest were selected for gene expression analysis in JEG-3 cells to investigate effects on expression of 46 genes involved in placental function.

## Methods

### Cell Culture

JEG-3 human choriocarcinoma trophoblastic cells (American Type Culture Collection, VA, United States) were maintained in sterile-filtered Minimum Essential Media (MEM) supplemented with l-glutamine and phenol red (Thermo Fisher Scientific, MA, United States), 10% (v/v) heat-inactivated BenchMark Fetal Bovine Serum (Gemini Bio, CA, United States), 1% (v/v) sodium pyruvate (Thermo Fisher Scientific, MA, United States), and 1% (v/v) penicillin streptomycin (Sigma Aldrich, MO, United States). Cells were maintained in an incubator at 37°C with 5% CO_2_. JEG-3 cells were cultured in 10 cm tissue culture plates and cell passage numbers were recorded.

### Experimental Design

A total of 42 perfluoroalkyl substances (PFAS) were evaluated (Supplementary Table S1 contains source and purity). PFAS stock solutions were maintained in 100% methanol as previous work has shown certain PFAS degrade when stored in dimethyl sulfoxide (DMSO; Gaballah et al., 2020). Each PFAS was tested across a 10-point linear concentration curve ranging from 50–500 µM (50 µM steps), except for perfluoroheptanesulfonic acid (CASRN 375-92-8; concentration curve of 23.25–232.5 µM [23.25 µM steps]) and 6:2 fluorotelomer phosphate diester (CASRN 57677-95-9; concentration curve of 37.5–375 µM [37.5 µM steps]), which were tested across compound-specific concentration curves due to solubility limitations. The vehicle control group consisted of 97.5% media + 2.5% methanol to match the concentration of methanol in the media at the highest PFAS concentration tested (500 µM). Menadione (50–500 µM) was used as a positive control for cytotoxicity assessment. PFAS were de-identified and assigned random numbers to eliminate bias. Chemical exposure plate maps were designed so that all experimental conditions were tested in at least technical duplicates per plate. The outer edges of each plate received media alone and were excluded from all analyses to avoid edge effects. Each plate map included 14 technical replicates of the vehicle control group and 14 technical replicates of media only. Biological replicates were defined as separate 384 well plates tested on separate days using different cell passages. The experimental workflow is depicted in Supplementary Figure S1.

JEG-3 cells were counted using a Z2 Coulter Particle Counter (Beckman Coulter, CA, United States) and seeded at a density of 2,500 cells per well in black-walled, clear bottom 384 well plates (Corning, #3764) and allowed to adhere overnight. Approximately 24 h later, 384 well plates were imaged using an IncuCyte Zoom live cell imager (Essen BioScience Inc., MI, United States) to obtain baseline cellular confluence measurements prior to chemical dosing (0 h). Media was removed from wells and replaced with fresh media containing chemical treatments (exposure media) using a Viaflo liquid handling device (Integra Biosciences, NH, United States). Within 30 min of exposure media application, plates were again imaged for cellular confluence using the IncuCyte Zoom. Plates were incubated with exposure media for 24 h and then imaged again using the IncuCyte Zoom (24 h).

### Proliferation

Plate well bottom images were evaluated using automated cell masking. The automated cell masking program was developed using IncuCyte Zoom base software. Cell masking parameters were defined using a training set of JEG-3 cell images obtained prior to conducting the experiments and following IncuCyte Zoom software recommendations. Briefly, the training images were obtained from cell culture plates with varying levels of JEG-3 cellular confluence (ranging from ∼15% confluent to ∼95% confluent). Parameters for the automated cell masking were first manually set by an experimenter for 3-6 images of low (∼15–30%), medium (∼30–70%), and high confluence (∼70% or greater). The training images were then evaluated by the automated masking program and manually checked to ensure the parameters were sufficiently strict to ignore background (e.g., blank space on the plate well bottom) but adequately sensitive to detect cell borders. The masking program was then implemented on the experimental plates to obtain a baseline cellular confluence measurement at 0 h and a second confluence measurement at 24 h.

Cell seeding density of 2,500 cells/well in 384 well plates was determined to result in a baseline confluence of ∼30–50% in order to capture cell proliferation in the linear phase of the growth curve between 0 and 24 h of the experiment. Cell proliferation was calculated as the difference between confluence at 24 h and baseline confluence at 0 h. Under control conditions, the absolute change in cell confluence over 24 h from baseline was ∼30% (e.g., plate well bottom confluence measurements increased from ∼40% at 0 h to ∼70% at 24 h).

### Mitochondrial Membrane Potential (MMP)

The JC-10 assay (Enzo Life Sciences, NY, United States) was used to evaluate mitochondrial membrane potential, the main bioenergetic parameter that regulates respiratory rate, ATP synthesis and reactive oxygen species generation. In cells with polarized mitochondrial membranes, the JC-10 dye localizes to the mitochondrial matrix and forms red fluorescent aggregates (540/590 nm). In cells with depolarized mitochondrial membranes, the JC-10 dye localizes in the cytoplasm and converts to a green fluorescent monomer (490/525 nm). Mitochondrial membrane potential was calculated as the ratio of the green and red fluorescence intensities within a well.

At the end of the 24 h exposure period, exposure media was removed from the plate. The plate wells were then washed with 1x phosphate buffered saline (1x PBS). After washing, the JC-10 reagent was suspended in fresh 1x PBS and 20 µl was added to each well at a final concentration of 20 μM (Enzo Life Sciences, NY, United States). The plate was incubated for 1 h at 37°C, and the fluorescence intensities at excitation/emission wavelengths of 490/525 nm and 540/590 nm were measured using a CLARIOstar (BMG Labtech, Offenburg, Germany) plate reader, respectively.

### Viability

In the same plate evaluated for mitochondrial membrane potential, cell viability measures were duplexed using the CellTiter-Glo (CTG) Luminescent Cell Viability Assay (Promega, WI, United States). CTG reagent was added to each well at a volume of 20 µl. The plate was left at room temperature to incubate for 5 min and then 20 µl from each well was transferred to a low-volume, round-bottom, white 384 well plate (Corning, NY, United States). The CTG reagent lyses cellular and mitochondrial membranes and binds to adenosine triphosphate (ATP) released by cells, which produces a luminescence signal. The intensity of the luminescence was considered a proxy measure for the number of live cells in a well. The luminescence intensities were measured using the CLARIOstar plate reader.

### Migration

JEG-3 cells were cultured as described in *Cell Culture* section, and six PFAS were evaluated across an 8-point linear concentration curve ranging from 25 to 200 µM (25 µM steps): PFOA (335-67-1), GenX (62037-80-3), PFOS (1763-23-1), perfluorooctanesulfonamide (PFOSA, 754-91-6), perfluorooctanamide (PFOAA, 423-54-1), and perfluorononanoic acid (PFNA, 375-95-1). The vehicle control group consisted of 98% media + 2% methanol to match the concentration of methanol in the media at the highest PFAS concentration tested (200 µM). Cadmium chloride (25 µM) was used as a positive control for cytotoxicity (Brooks and Fry, 2017). Chemical exposure plate maps were designed so that all experimental conditions were tested in at least technical duplicates per plate. The outer edges of each plate received media alone and were excluded from all analyses to avoid edge effects. JEG-3 cells were counted and seeded at a density of 80,000 cells per well in black-walled, clear bottom 96-well plates (Corning #CLS3603, Millipore Sigma) and allowed to adhere and grow for 36 h. After approximately 36 h, 96-well plates were imaged on the IncuCyte Zoom to obtain cellular confluence measurements prior to chemical dosing (0 h). Media was then removed from wells and replaced with fresh serum-free media containing chemical treatments (serum-free exposure media) using the Viaflo liquid handling device. After 1 h of treatment with serum-free exposure media, plates were again imaged for cellular confluence using the IncuCyte Zoom. Serum-free exposure media was then removed from wells and wells were scratched by dragging a 200 µl pipette tip from top to bottom of each well, changing tips every well. Fresh media containing serum and chemical treatments (exposure media) was added to plates using a liquid handling device and allowed to grow for 72 h, with imaging via the IncuCyte Zoom every 6 h.

Plate well bottom images were evaluated using automated cell masking as described in *Proliferation* section. Cell migration was calculated as the change in cellular confluence from the baseline measurement obtained immediately post-scratch to each subsequent time point (24, 48, and 72 h, post-scratch).

### Gene Expression

JEG-3 cells were seeded at a density of 80,000 cells per well in six well plates and allowed to adhere overnight (24 h). The following day, media was removed and replaced with exposure media, then returned to the incubator for 24 h. The test compounds included non-cytotoxic concentrations of PFOA (0, 1, and 100 µM) and GenX (0, 3, 300 µM). These test compounds were selected *a priori* due to their high public health relevance. Each 6 well plate contained the three concentrations for either PFOA or GenX, with each condition in technical duplicate. Plates containing these PFOA or GenX exposure conditions were replicated three times (N = 3). After 24 h, exposure media was aspirated and cells were washed in ice cold 1xPBS, which was then aspirated. Each well received 500 µl of TRIzol reagent (Thermo Fisher Scientific, MA, United States) and cells were harvested in the TRIzol using a cell scraper. Technical duplicate wells within a plate were combined to generate a single sample of ∼1,000 µl. RNA was isolated from the resulting cellular homogenate using RNeasy Mini Kits (Qiagen, Hilden, Germany). Isolated RNA quality was validated and concentration determined using a NanoDrop 2000 (Thermo Fisher Scientific, MA, United States). 100 ng of mRNA from each sample was then analyzed using a custom gene expression code set (NanoString, Seattle, WA, United States). The custom code set included 46 genes selected for their downstream involvement in placental development (e.g., *WNT4*), endocrine signaling (e.g., *CYP19A1*), fetal growth/nutrient sensing (e.g., *IGF2*), inflammation (e.g., *IL6*), oxidative stress (e.g., *SOD1, GPX1*), and xenobiotic transport/metabolism (e.g., *ABCG2*, *MRP3*).

### Data Processing and Statistical Analysis

HTTS data were analyzed using a custom data analysis script in R v 1.2.5019 (R Core Team, 2019). Briefly, raw data points corresponding to the confluence measurements at 0 and 24 h, the fluorescence intensities corresponding to MMP, and the relative luminescence units (RLU) corresponding to cell viability were imported directly from Microsoft Excel into the R programming environment, RStudio. Separate plates run on separate days using different JEG-3 passages were considered as biologic replicates. Technical replicates within a plate were first evaluated as pseudoreplicates, but this precluded robust concentration-response model fits thus technical replicates of experimental values within a plate were considered as independent data points. After excluding plate edges, all remaining data points were preprocessed by eliminating wells with confluence values <20% and >80% at time 0 h. Cellular proliferation was calculated by subtracting the cellular confluence at 0 h from the cellular confluence at 24 h to yield change in confluence (Δ confluence). MMP was calculated as the ratio of red:green fluorescence intensities.

Prior to evaluating experimental data, data obtained from control wells (wells exposed to media only and wells exposed to 2.5% (v/v) methanol) were evaluated for quality control. Control wells were examined for variability in response within and between plates. Average confluence at 0 h was compared across control conditions and plates. Plates that passed quality control were included in subsequent data analyses and plates that failed to pass quality control were rejected.

Preprocessed raw data points were then transformed to relative values based on the mean of control wells within in a plate using the following formula: [experimental value/average (all control values)] × 100. These relative values were then used to perform concentration-response modeling using the *bmd* package (Ritz et al., 2015). All endpoints were fit to four parameter concentration-response curves without constraints. Models were fit to each endpoint (proliferation, MMP, and viability) for each PFAS tested. Model estimates were reviewed manually and model fits were accepted if the following criteria were met: 1) estimated EC50 was within the range of the tested concentration-response curve (50–500 µM), 2) standard deviations no larger than a doubling of the EC50 estimate itself, and 3) the concentration-response model did not fail to converge (e.g., no “NaN” or “NA” values in R output). EC50 values that met these criteria were extracted and used in subsequent data categorizations and visualizations. Raw model output values including those that did not meet inclusion criteria are shown in Supplementary Table S2.

Data were also analyzed using predetermined statistical cutoffs (referred to here as “rough binning”) to further categorize individual compounds by activity and to allow examination of individual concentrations tested. For all endpoints, vehicle control normalized values corresponding to each concentration tested for each congener were averaged within and between biological replicates to produce a single value for each concentration tested per individual compound (e.g., 10 experimental concentrations resulted in a total of 10 data points per congener per endpoint). The values were then categorized based on deviance from the range of normalized vehicle control values (e.g., normalized test value > normalized vehicle control mean + 2*normalized vehicle control standard deviation or normalized test value < normalized vehicle control mean - 2*normalized vehicle control standard deviation). This method allowed for the identification of compounds that may perturb endpoints above or below the expected range of normal depending on concentration, allowing for identification of potential nonmonotonic concentration-response relationships precluded by 4-parameter concentration-response modeling.

Raw migration assay data exported from IncuCyte software were analyzed using GraphPad Prism 8.0 (GraphPad Software, San Diego, CA)). Prior to analysis, a minimum starting confluence value of 70% was set to ensure comparisons between samples were not skewed. Images with a starting confluence of less than 70% were excluded from analysis. Statistical analysis was performed using multiple unpaired t-tests to compare treated groups to the vehicle control followed by *post hoc* correction for multiple tests using the Bonferroni-Dunn method. Concentration-response analysis to determine migration assay EC50s was attempted but was not able to be modeled due to the non-linear nature of the concentration-response relationship for this endpoint.

Gene expression data were quantified using the nCounter Digital Analyzer (NanoString, Seattle, WA, United States) and raw and adjusted counts were generated with nSolver (v4.0) software (NanoString, Seattle, WA, United States). Data were adjusted utilizing the manufacturer’s positive and negative experimental control probes, as well as housekeeping genes (*ACTA2*, *ACTB*, *B2M*, *LDHA*, *RNA18S5*, and *ROLYR2L*). All samples passed nSolver’s initial QA/QC controls and replicates were very well correlated with *R* ^2^ values greater than 0.99. Adjusted data were then imported into Partek v7.0 (St. Louis, MO, United States), log2 transformed and quantile normalized for further QA/QC and statistical analyses. Genes with mRNA counts below the detection threshold of 20 were removed due to suboptimal probe hybridization (N = 2 genes for GenX; *GH2* and *IGFBP5*). Fold change values were analyzed by ANOVA with *post hoc* false discovery rate adjustment (FDR) of *p* < 0.1. Normalized gene expression values were visualized using *pheatmap* with Euclidean clustering of genes and concentration groups (Kolde and Kolde, 2015).

## Results

Overall, concentration-response modeling of PFAS effects on JEG-3 cell viability, proliferation, and MMP resulted in quantification of an EC50 value that met inclusion criteria for at least one of the three evaluated endpoints for 33 of 42 compounds (79%; Table 1). EC50 values were obtained for all three endpoints for 13 PFAS (31%), for two of three endpoints for 15 PFAS (36%), for one of three endpoints for five PFAS (12%), and zero of three endpoints for nine PFAS (21%; Table 1). Cells exposed to vehicle control (methanol 2.5% v/v in media) did not differ from cells exposed to media only in either cellular morphology or viability after 24 h (Supplementary Figure S2). The positive control for cell death, menadione, was not cytotoxic at 100 μM, but promoted cell death at ≥150 μM (shown in Supplementary Figure S3). In general, there was good agreement between concentration-response model-derived EC50 estimates and the “rough binning” results, which examined effects of PFAS on the HTTS endpoints on a concentration-by-concentration basis, thus allowing for detection of potential non-monotonic effects in the absence of a robust concentration-response model fit. For example, EC50 values were not able to be derived for GenX (62037-80-3) for any HTTS endpoint due to poor model fit (Table 1), but the rough binning method identified 350 and 50 µM as the lowest concentrations that significantly decreased viability and proliferation, respectively (Supplementary Table S2). For PFAS with robust model fits, rough binning results were highly consistent with estimated EC50s. For example, the EC50 estimates and accompanying rough binned results for PFOS (1763-23-1) were 291 and 300 µM for viability, 294 and 250 µM for proliferation, and 353 and 350 µM for MMP, respectively (Table 1 and Supplementary Table S2).

**TABLE 1:** Dose-response curve EC50 estimates for JEG-3 cell viability, proliferation, and mitochondrial membrane potential (MMP) after 24 h exposure to PFAS.

Name	CASRN	Viability EC50 ± SD (µM)	Proliferation EC50 ± SD (µM)	MMPEC50 ± SD (µM)
Perfluoro-2-methyl-3-oxahexanoic acid	13252-13-6	*nc*	*nc*	*nc*
Perfluoro-3,6-dioxadecanoic acid	137780-69-9	*nc*	282.8 ± 25.7	350.1 ± 16.1
8-H-Perfluorooctanoic acid	13973-14-3	317.5 ± 182.9	441.3 ± 18.1	147.3 ± 27.1
Perfluoro-3,6-dioxaheptanoic acid	151772-58-6	326.5 ± 85.7	351.3 ± 11.8	368.1 ± 22.6
Perfluoro-3,6,9-trioxadecanoic acid	151772-59-7	267.2 ± 26.8	302.9 ± 30.1	*nc*
7H-Perfluoroheptanoic acid	1546-95-8	222.8 ± 60.2	294.4 ± 48.2	*nc*
Perfluorooctanesulfonic acid	1763-23-1	291.2 ± 9.1	294.2 ± 5.8	352.9 ± 6.7
Perfluoroundecanoic acid	2058-94-8	266.1 ± 12.4	229.5 ± 13.3	200.1 ± 4.0
2,2-Difluoro-2-(trifluoromethoxy)acetate sodium salt	21837-98-9	*nc*	*nc*	*nc*
Perfluoropentanoic acid	2706-90-3	*nc*	*nc*	174.7 ± 68.4
6:2 Fluorotelomer sulfonic acid	27619-97-2	*nc*	*nc*	*nc*
2-(N-Ethylperfluorooctanesulfonamido)acetic acid	2991-50-6	202.7 ± 44.7	203.6 ± 17.7	*nc*
Perfluorobutylsulfonamide	30334-69-1	*nc*	*nc*	*nc*
Perfluorohexanoic acid	307-24-4	*nc*	*nc*	142.4 ± 12.6
Perfluorododecanoic acid	307-55-1	361.1 ± 30.8	317.8 ± 24.5	*nc*
Perfluoro-3,6,9-trioxatridecanoic acid	330562-41-9	194.9 ± 54.6	332.4 ± 34.1	*nc*
Perfluorooctanoic acid	335-67-1	357.7 ± 16.9	344.1 ± 12.2	360.2 ± 18.0
Perfluorodecanoic acid	335-76-2	180.7 ± 25.6	234.0 ± 22.8	235.8 ± 159.6
Perfluorohexanesulfonic acid	355-46-4	288.7 ± 29.6	251.6 ± 21.7	86.6 ± 71.5
Perfluorobutanoic acid	375-22-4	226.0 ± 115.7	209.2 ± 359.8	*nc*
Perfluorobutanesulfonic acid	375-73-5	330.4 ± 35.0	223.2 ± 76.7	*nc*
Perfluoroheptanoic acid	375-85-9	486.7 ± 408.6	277.4 ± 81.6	*nc*
Perfluoroheptanesulfonic acid	375-92-8	*nc*	296.2 ± 201.3	*nc*
Perfluorononanoic acid	375-95-1	332.6 ± 10.3	326.1 ± 5.5	304.6 ± 13.6
5H-Octafluoropentanoic acid	376-72-7	353.7 ± 28.6	333.7 ± 56.5	*nc*
Perfluoro-3-methoxypropanoic acid	377-73-1	303.7 ± 28.2	204.1 ± 110.9	93.1 ± 131.6
8:2 Fluorotelomer sulfonic acid	39108-34-4	159.5 ± 45.7	321.3 ± 171.2	331.1 ± 5.7
Perfluorooctanamide	423-54-1	*nc*	114.0 ± 35.6	294.6 ± 66.1
6:2 Fluorotelomer phosphate diester	57677-95-9	*nc*	141.8 ± 39.2	*nc*
6:2 Fluorotelomer phosphate monoester	57678-01-0	182.7 ± 21.7	193.3 ± 3.8	248.0 ± 62.1
8:2 Fluorotelomer phosphate monoester	57678-03-2	359.8 ± 30.3	250.7 ± 37.6	312.5 ± 29.8
Menadione (positive control)	58-27-5	205.5 ± 47.1	157 ± 21.4	*nc*
Ammonium perfluoro-2-methyl-3-oxahexanoate	62037-80-3	*nc*	*nc*	*nc*
Sodium perfluoropentanesulfonate	630402-22-1	*nc*	*nc*	*nc*
6:2 Fluorotelomer alcohol	647-42-7	415.4 ± 70.6	*nc*	332.4 ± 44.1
8:2 Fluorotelomer alcohol	678-39-7	35.7 ± 36.5	*nc*	95.1 ± 15.3
8:2 Fluorotelomer phosphate diester	678-41-1	*nc*	*nc*	*nc*
Perfluorotridecanoic acid	72629-94-8	*nc*	388.1 ± 124.9	*nc*
Perfluorooctanesulfonamide	754-91-6	175.9 ± 4.9	160.0 ± 87.8	227.4 ± 369.9
9-H-Perfluorononanoic acid	76-21-1	349.9 ± 43.1	*nc*	186.9 ± 27.6
Perfluoro (4-methoxybutanoic) acid	863090-89-5	*nc*	*nc*	*nc*
1H,1H-Nonafluoropentyl p-toluenesulfonate	883499-79-4	*nc*	306.1 ± 47.9	158.6 ± 17.7
2H,2H,3H,3H-Perfluorooctanoic acid	914637-49-3	*nc*	*nc*	*nc*

Note: nc, not calculable.

### Viability

**FIGURE 1:**
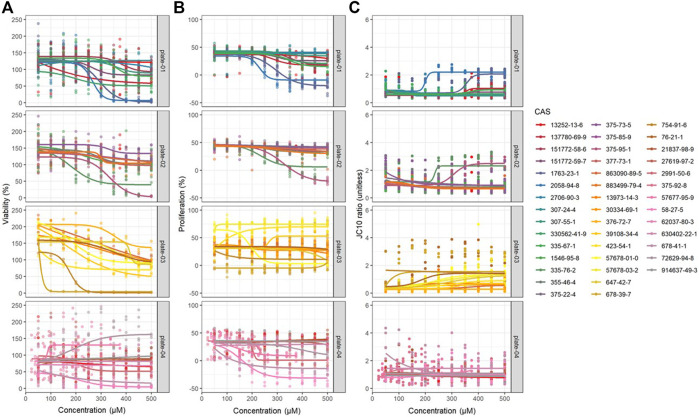
Dose-response modeling results obtained from JEG-3 cells exposed to 42 different PFAS congeners for 24 h corresponding to **(A)** viability, **(B)** proliferation, and **(C)** mitochondrial membrane potential (MMP). Data were fit to a four-parameter dose-response model with no constraints and EC50 estimates were extracted. *N* = 3 biological replicates.

**FIGURE 2:**
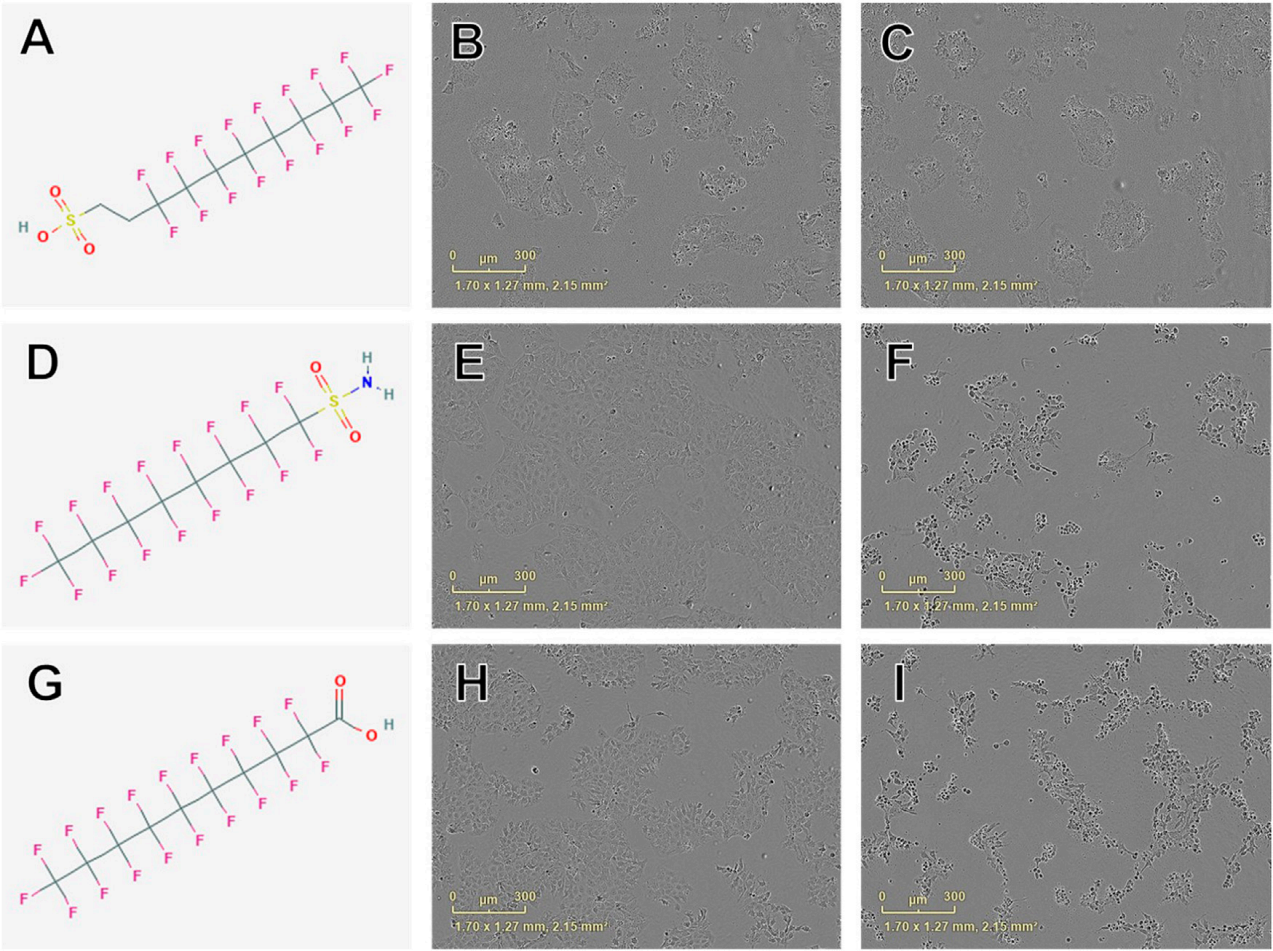
Examples of chemical structures and corresponding phase contrast live cell images obtained from JEG-3 cells after 24 h exposure to PFAS with most pronounced effects on cellular viability. **(A)** Structure of 8:2 fluorotelomer sulfonic acid (39108-34-4; viability EC50 ± SE: 160 ± 46 µM); **(B)** Image of cells exposed to 300 µM 39108-34-4; **(C)** Image of cells exposed to 500 µM 39108-34-4; **(D)** Structure of perfluorooctanesulfonamide (754-91-6; EC50 ± SE: 176 ± 5 µM); **(E)** Image of cells exposed to 150 µM 754-91-6; **(F)** Image of cells exposed to 300 µM 754-91-6; **(G)** Structure of perfluorodecanoic acid (335-76-2; EC50 ± SE: 181 ± 26 µM); **(H)** Image of cells exposed to 250 µM 335-76-2; **(I)** Image of cells exposed to 350 µM 335-76-2. Mild to moderate cell stress is apparent in **(B)**, **(E)**, and **(H)** marked by darkened, condensed nuclei and increased fibroblastic projections. Moderate to severe cell stress and death is apparent in **(C)**, **(F)**, and **(I)**.

Concentration-response curve modeling provided viability EC50 estimates for 27 of 42 PFAS (66%) that met EC50 criteria (Table 1 and Figure 1). Cell viability EC50 values ranged from 35.7 µM (678-39-7) to 486.7 µM (375-85-9; Table 1). Of PFAS from which a viability EC50 estimate was calculated, the five compounds with the lowest EC50 ± SE were: 8:2 Fluorotelomer alcohol (678-39-7; 36 ± 37 µM), 8:2 Fluorotelomer sulfonic acid (39108-34-4; 160 ± 46 µM), perfluorooctane sulfonamide (754-91-6; 176 ± 5 µM), perfluorodecanoic acid (335-76-2; 181 ± 26 µM), 6:2 Fluorotelomer phosphate monoester (57678-01-0; 183 ± 22 μM; Table 1 and Supplementary Figure S4). Examples of structures and live cell images are shown in Figure 2.

Rough binning (as described in *Data Processing and Statistical Analysis* section) of test compounds resulted in 10 PFAS (24%) that reduced cell viability relative to vehicle control treatment after 24 h in at least one concentration: Perfluoro-3,6-dioxadecanoic acid (137780-69-9), perfluorooctane sulfonic acid (1763-23-1), perfluoro-3,6,9-trioxatridecanoic acid (151772-59-7), perfluoroundecanoic acid (2058-94-8), perfluorodecanoic acid (335-76-2), perfluorononanoic acid, 2-(N-ethylperfluorooctanesulfonamido) acetic acid (2991-50-6), 8:2 fluorotelomer phosphate diester (678-41-1), ammonium perfluoro-2-methyl-3-oxahexanoate (62037-80-3), and sodium perfluoropentanesulfonate (630402-22-1; Supplementary Table S3). Menadione control reduced cell viability relative to vehicle control treatment using rough binning (Supplementary Figure S4).

A total of 13 PFAS (31%) increased cell viability in at least one concentration relative to vehicle control: Perfluoro-3,6-dioxaheptanoic acid (151772-58-6), perfluorododecanoic acid (307-55-1), perfluorohexanoic acid (307-24-4), perfluoropentanoic acid (2706-90-3), 1H,1H-nonafluoropentyl p-toluenesulfonate (883499-79-4), 7H-perfluoroheptanoic acid (1546-95-8), perfluorobutanesulfonic acid (375-73-5), perfluorobutanoic acid (375-22-4), perfluorodecanoic acid (335-76-2), perfluorohexanesulfonic acid (355-46-4), 6:2 fluorotelomer phosphate diester (678-41-1), perfluorotridecanoic acid (72629-94-8), and 2H,2H,3H,3H-perfluorooctanoic acid (883499-79-4; Supplementary Table S3).

### Proliferation

**FIGURE 3:**
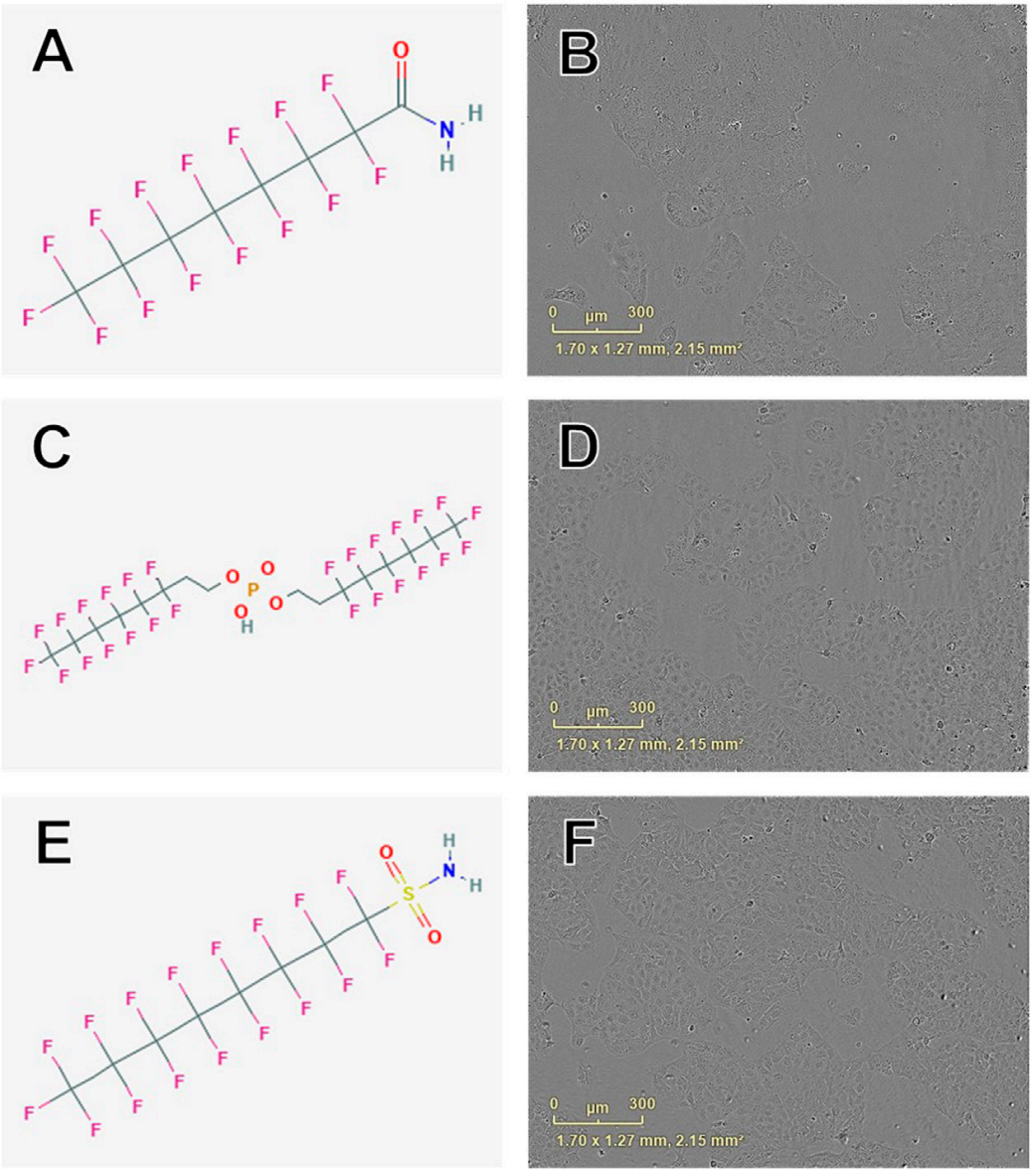
Examples of chemical structures and corresponding phase contrast live cell images obtained from JEG-3 cells after 24 h exposure to PFAS with most pronounced effects on cellular proliferation. **(A)** Structure of perfluorooctanamide (423-54-1; EC50 ± SE: 114.0 ± 35.6 µM); **(B)** Live cell image of cells exposed to 150 µM 423-54-1; **(C)** Structure of 6:2 fluorotelomer phosphate diester (57677-95-9; EC50 ± SE: 141.6 ± 39.4 µM); **(D)** Image of cells exposed to 150 µM 57677-95-9; **(E)** Structure of perfluorooctane sulfonamide (754-91-6; EC50 ± SE: 159.8 ± 90.0 µM); **(F)** Image of cells exposed to 150 µM 754-91-6. No overt cell death is apparent in **(B)**, **(D)**, or **(F)**.

Concentration-response curve modeling provided proliferation EC50 estimates for 28 of 42 PFAS (68%) that met EC50 criteria (Table 1 and Figure 1). Cell proliferation EC50 values ranged from 114.0 µM (perfluorooctanamide, 423-54-1) to 441.3 µM (8-H-perfluorooctanoic acid, 13973-14-3; Table 1). Of PFAS from which a proliferation EC50 estimate was calculated, the five compounds with the lowest EC50 ± SE were: Perfluorooctanamide (423-54-1; 114.0 ± 35.6 µM), 6:2 fluorotelomer phosphate diester (57677-95-9; 141.6 ± 39.4 µM), perfluorooctane sulfonamide (754-91-6; 159.8 ± 90.0 µM), 6:2 Fluorotelomer phosphate monoester (57678-01-0; 193 ± 4 µM), and 2-(N-Ethylperfluorooctanesulfonamido)acetic acid (2991-50-6; 204 ± 18 μM; Table 1 and Supplementary Figure S4). Examples of chemical structures and live cell phase contrast images are shown in Figure 3.

Rough binning (as described in *Data Processing and Statistical Analysis* section) of test compounds resulted in 19 PFAS (45%) that reduced cellular proliferation relative to vehicle control treatment in at least one concentration: Perfluoro-3,6-dioxadecanoic acid (137780-69-9), perfluoro-3,6-dioxaheptanoic acid (151772-58-6), perfluoro-3,6,9-trioxadecanoic acid (151772-59-7), perfluorooctanesulfonic acid (1763-23-1), perfluoro-3,6,9-trioxatridecanoic acid (330562-41-9), perfluorohexanoic acid (307-24-4), perfluoroundecanoic acid (2058-94-8), 1H,1H-nonafluoropentyl p-toluenesulfonate (883499-79-4), 7H-perfluoroheptanoic acid (1546-95-8), perfluorodecanoic acid (335-76-2), perfluorooctanoic acid (335-67-1), perfluoroheptanoic acid (375-85-9), perfluorohexanesulfonic acid (355-46-4), perfluorononanoic acid, 2-(N-ethylperfluorooctanesulfonamido) acetic acid (2991-50-6), 6:2 fluorotelomer phosphate diester (678-41-1), 8:2 fluorotelomer phosphate diester (678-41-1), perfluoroheptanesulfonic acid (375-92-8), and perfluorotridecanoic acid (72629-94-8; Supplementary Table S3). Menadione control reduced proliferation relative to vehicle control treatment using rough binning.

Rough binning (as described in *Data Processing and Statistical Analysis* section) of test compounds resulted in 10 PFAS (24%) that increased cellular proliferation relative to vehicle control treatment in at least one concentration: Perfluoro-2-methyl-3-oxahexanoic acid (13252-13-6), 2-(N-ethylperfluorooctanesulfonamido) acetic acid (13252-13-6), 2,2-difluoro-2-(trifluoromethoxy)acetate sodium salt (21837-98-9), 2H,2H,3H,3H-perfluorooctanoic acid (914637-49-3), 6:2 fluorotelomer phosphate diester (678-41-1), 6:2 fluorotelomer sulfonic acid (27619-97-2), ammonium perfluoro-2-methyl-3-oxahexanoate (62037-80-3), perfluoroheptanesulfonic acid (375-92-8), perfluorotridecanoic acid (72629-94-8), and sodium perfluoropentanesulfonate (630402-22-1; Supplementary Table S3).

### Mitochondrial Membrane Potential

**FIGURE 4:**
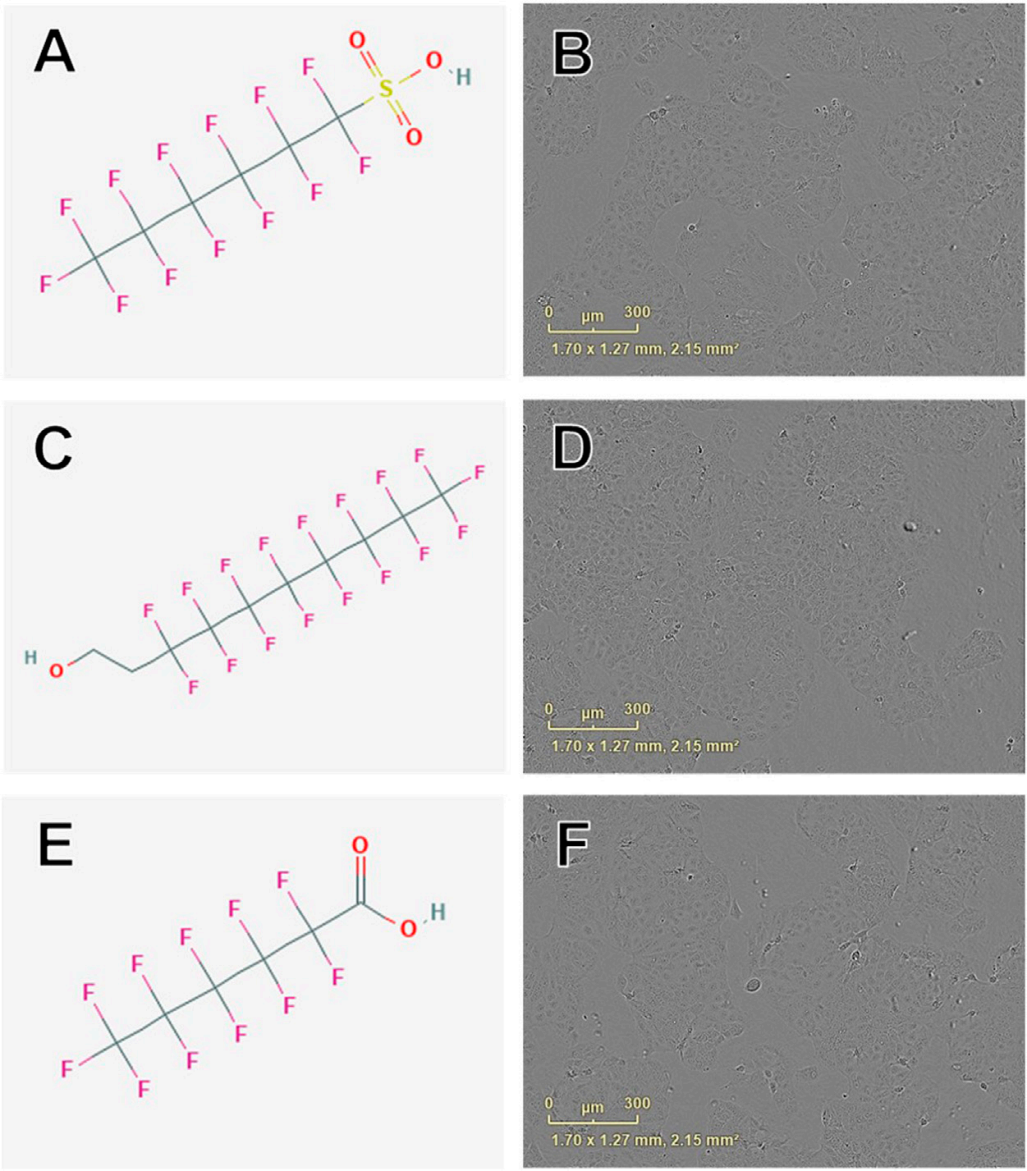
Examples of chemical structures and corresponding phase contrast live cell images obtained from JEG-3 cells after 24 h exposure to PFAS with most pronounced effects on mitochondrial membrane potential. **(A)** Structure of perfluorohexanesulfonic acid (355-46-4; EC50 ± SE: 86.2 ± 72.3 µM); **(B)** Live cell image of cells exposed to 150 µM 355-46-4; **(C)** Structure of 6:2 fluorotelomer alcohol (678-39-7 EC50 ± SE: 95.4 ± 17.1 µM); **(D)** Image of cells exposed to 50 µM 678-39-7; **(E)** Structure of perfluorohexanoic acid (307-24-4; EC50 ± SE: 140.9 ± 11.10 µM); **(F)** Image of cells exposed to 150 µM 307-24-4. No overt cell death is apparent in **(B)**, **(D)**, or **(F)**.

Concentration-response curve modeling provided MMP EC50 estimates for 19 of 42 PFAS (45%) that met EC50 criteria (as described in *Data Processing and Statistical Analysis* section) are shown in Table 1. MMP EC50 values ranged from 86.2 µM (perfluorohexanesulfonic acid; 355-46-4) to 368.0 µM (perfluoro-3,6-dioxaheptanoic acid; 151772-58-6). Of PFAS from which a MMP EC50 estimate was calculated, the five compounds with the lowest EC50 ± SE were: Perfluorohexanesulfonic acid (355-46-4; 86.2 ± 72.3 µM), 6:2 fluorotelomer alcohol (678-39-7; 95.1 ± 15.3 µM), perfluorohexanoic acid (307-24-4; 140.9 ± 11.1 µM), 8-H-perfluorooctanoic acid (13973-14-3; 147.3 ± 27.1 µM), and 1H,1H-Nonafluoropentyl p-toluenesulfonate (883499-79-4; 158.6 ± 17.7 µM; Table 1 and Supplementary Figure S6). Examples of chemical structures and live cell phase contrast images are shown in Figure 4.

Rough binning (as described in *Data Processing and Statistical Analysis* section) of compounds by reduced MMP relative to vehicle control treatment after 24 h in at least one concentration resulted in 15 PFAS (35%): Perfluoro-2-methyl-3-oxahexanoic acid (13252-13-6), perfluoro-3,6-dioxadecanoic acid (137780-69-9), perfluoro-3,6-dioxaheptanoic acid (151772-58-6), perfluoro-3,6,9-trioxadecanoic acid (151772-59-7), perfluorooctanesulfonic acid (1762-23-1), perfluro-3,6,9-trioxatridecanoic acid (330562-41-9), perfluorohexanoic acid (307-24-4), perfluoroundecanoic acid (2058-94-8), perfluorobutanesulfonic acid (375-73-5), perfluorobutanoic acid (375-22-4), perfluorooctanoic acid (335-67-1), perfluorodecanoic acid (335-76-2), perfluorononanoic acid (375-95-1), 2-(N-ethylperfluorooctanesulfonamido) acetic acid (2991-50-6), and 8:2 fluorotelomer phosphate diester (678-41-1; Supplementary Table S3). Menadione control reduced MMP relative to vehicle control treatment after 24 h using rough binning. Rough binning of compounds by increased MMP relative to vehicle control treatment after 24 h in at least one concentration resulted in 0 PFAS (0%).

### Migration

Six PFAS were selected for the migration assay: PFOA (335-67-1), GenX (62037-80-3), PFOS (1763-23-1), PFOSA (754-91-6), PFOAA (423-54-1), and PFNA (375-95-1). These PFAS were selected due to their public health relevance as an emerging (e.g., GenX) or legacy chemical (e.g., PFOA, PFOS, PFNA) and/or bioactivity in the HTTS (e.g., PFOSA, which exhibited low EC50 values in both the viability and proliferation assays and PFOAA, which exhibited the lowest EC50 in the proliferation assay).

**FIGURE 5:**
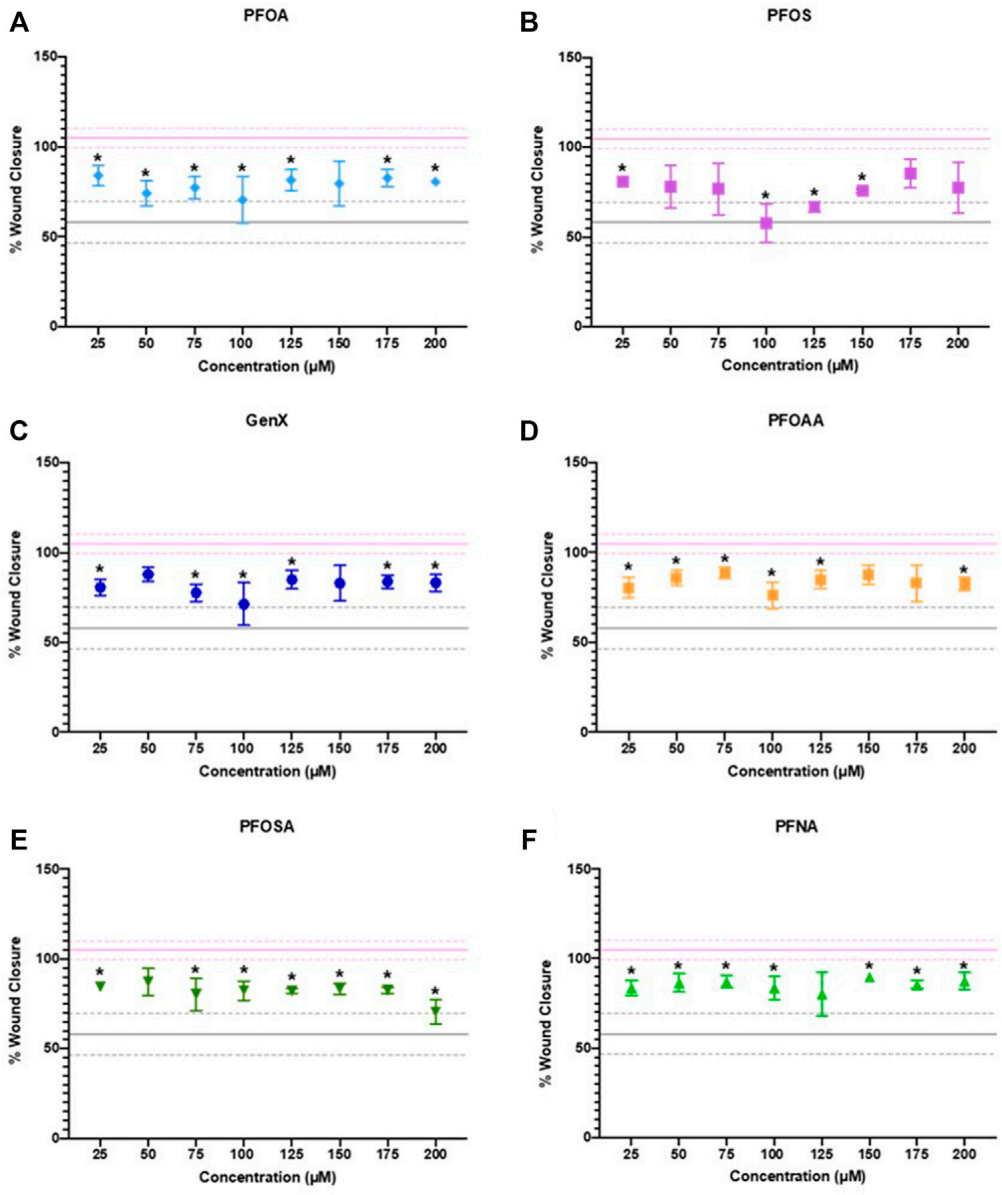
JEG‐3 cell migration after 24 h of exposure to **(A)** PFOA, **(B)** PFOS, **(C)** GenX, **(D)** PFOAA, **(E)** PFOSA, and **(F)** PFNA, assessed using a scratch wound assay. For each chemical, migration is expressed as % wound closure, which measures the extent to which JEG‐3 cells migrated across the scratch wound between the initial scratch was made and 24 h post-scratch (mean ± SD). The upper pink solid line flanked by dotted lines represents the mean ± SD for vehicle control (2% methanol). The lower gray solid line flanked by dotted lines represents the mean ± SD values for the positive control (25 µM cadmium chloride). N = 2−4 biological replicates per chemical and dose. Significance was determined using multiple unpaired t‐tests comparing experimental conditions to the vehicle control followed by post hoc correction using the Bonferroni-Dunn method, *p < 0.05. Representative images are shown in Supplementary Figure S7.

After 24 h of exposure, cellular migration was significantly reduced by exposure to PFOA, PFOS, GenX, PFOAA, PFOSA, and PFNA, relative to vehicle control (Figure 5). All six PFAS significantly reduced cellular migration, but the concentrations at which significant effects were detected was not uniform across the chemicals and was not a linear concentration-response. Representative images of cell migration over the experimental time course for each chemical are shown in Supplementary Figure S7. Cellular migration was also evaluated at 48 and 72 h, although the significant effects of any concentration of PFOA or PFNA observed at 24 h were attenuated by 72 h (Supplementary Figures S8, S9). It should be noted that reduced migration at the higher end of the concentration curve for PFOSA may have been influenced by cytotoxic effects for this chemical (EC50 of 175 ± 4.9 µM). Other responses were well under the cytotoxic range for those individual chemicals.

### Differential Gene Expression Analysis

**FIGURE 6:**
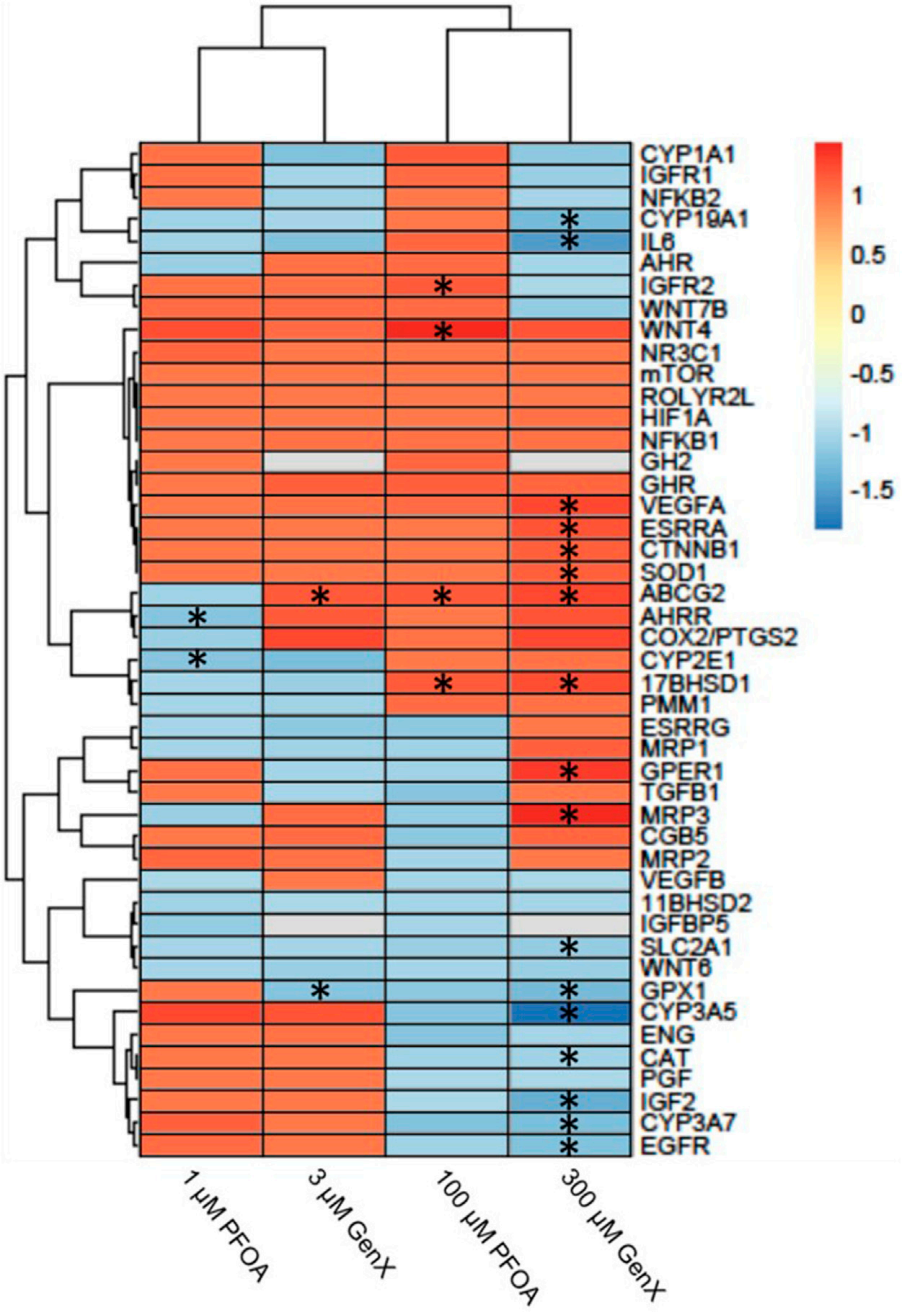
Heatmap illustrating gene expression changes in a set of 46 genes evaluated in JEG‐3 cells after exposure to PFOA or GenX for 24 h. Fold‐change is indicated by the scale bar and colors on the heatmap, with grey indicating genes with mRNA counts below the detection threshold due to suboptimal probe hybridization (*GH2* and *IGFBP5*). Experimental group fold‐change values were compared to corresponding vehicle control group fold‐change values by ANOVA. Asterisks indicate significant shifts in gene expression at a false discovery rate of *p < 0.05 (N = 3 biological replicates).

After 24 h of exposure to PFOA at 1 or 100 µM or GenX at 3 or 300 μM, 21 genes (46%) with significantly altered gene expression were identified in one or more of the treatment groups (FDR *p* < 0.1; Figure 6 and Supplementary Table S4). Gene expression was most affected after exposure to 300 µM GenX (N = 15 genes or 32%, Figure 6 and Supplementary Table S4). Gene expression was also significantly altered after exposure to 1 µM PFOA (*AHRR* & *CYP2E1*), 100 µM PFOA (*17BHSD1, ABCG2, IGFR2,* & *WNT4*), or 3 µM GenX (*ABCG2* & *GPX1*; Figure 6 and Supplementary Table S4).

## Discussion

We evaluated the effect of exposure to 42 unique PFAS on cell viability, proliferation, and mitochondrial membrane potential and a subset of six PFAS on cell migration in human-derived placental JEG-3 trophoblasts. Implementation of this multiplexed high throughput toxicity screen resulted in a robust dataset from which concentration-response model estimates could be extracted for at least one endpoint in roughly 80% of evaluated PFAS. This experimental platform provides a rapid method for simultaneous evaluation of multiple PFAS compounds across three biologically relevant endpoints. To our knowledge, 34 of the 42 PFAS evaluated here have never been investigated for effects on placental cell viability, proliferation, or mitochondrial membrane potential. Thus, this work provides novel toxicity data for multiple PFAS. Furthermore, we investigated the effect of sub-cytotoxic exposure to two PFAS of high public health relevance, PFOA and GenX, on expression of 46 genes in JEG-3 cells and report altered expression of genes involved in xenobiotic transport, endocrine function, and inflammation/oxidative stress. This builds upon our prior work that demonstrated adverse placental outcomes in mice after *in vivo* gestational exposure to PFOA or GenX (Blake et al., 2020).

Unlike previously reported data in other cell lines (Buhrke et al., 2013), we did not observe a consistent relationship between carbon chain length and cell viability. Additionally, EC50 estimates for JEG-3 cell viability reported in our study for PFOA, PFOS, PFNA, and PFDoA were not consistent with those reported by Gorrochategui et al. (2014); however, these differences could potentially be explained by differences in experimental methods, such as the utilization of different cell viability assays and different concentration-response curves (linear vs. logarithmic) (Gorrochategui et al., 2014). While certain PFAS evaluated in this screen exhibited strong concentration-response relationships that fit a traditional concentration-response model (e.g., PFOS, PFOA, PFNA), there were multiple compounds that did not exhibit a clear concentration-response relationship (e.g., GenX, perfluoropentanoic acid, perfluorohexanoic acid). A strength of this work is the combination of implementing concentration-response modeling to derive EC50s in tandem with a concentration-by-concentration evaluation of PFAS effects on the HTTS endpoints. This approach allowed for the detection of potentially non-concentration-responsive effects due to PFAS exposure as well as building confidence in the platform due to the high degree of agreement between EC50 values and “rough binning” results. Future development of HTTS platforms for PFAS should consider and test for the possibility of non-linear and non-concentration-responsive relationships.

To our knowledge, this work describes the first PFAS HTTS using a placental cell line, though prior work has leveraged other cell lines in PFAS HTTS efforts. A subclone of human hepatocellular carcinoma-derived HepG2 cells expressing elevated CYP have been used to test 142 unique PFAS for 81 different transcription factor activities (Houck et al., 2021). This work similarly utilized a 24-h exposure period with exposure concentrations ranging from 0.137 to 300 µM and demonstrated nuclear receptor activation at concentrations lower than 1 µM in some cases (Houck et al., 2021). Taken together with the gene expression results reported here at the 1 and 3 µM exposure level, these data suggest PFAS exposure can initiate molecular events at concentrations considerably lower than those required to perturb functional endpoints at the level of the cell (e.g., migration). Future work should explore lower PFAS concentrations in placental cell lines and investigate molecular changes.

Relating *in vitro* exposure conditions to those tested in *in vivo* systems and detected in human exposure biomonitoring and epidemiological studies is important for interpreting *in vitro* data. Here we tested a concentration range of 50-500 µM in the HTTS, 25-200 µM in the migration assay, and 1-300 µM in the gene expression analysis, which corresponds to concentration ranges of 10.1–495.1, 10.3–100.0, and 0.41–99.0 μg/ml, respectively. These concentration ranges overlap with concentrations of PFAS reported in serum of people in highly contaminated communities (PFOA: 17.6 μg/ml; Steenland et al., 2009). PFAS concentrations reported in the serum of pregnant women (PFOS in the 95th percentile of exposure = 0.018 μg/ml; Bommarito et al., 2021) and concentrations measured in human placental samples (maximum PFHxA = 0.006 μg/g; Bangma et al., 2020b) are within two to four orders of magnitude of the concentrations tested in the present work. Notably, Bommarito et al. (2021) reported maternal PFOS exposure was associated with higher odds of late-onset preeclampsia (OR = 1.60, 95% CI: 1.06, 2.43). Cytotoxicity EC50 values reported in the current work range from 16.6–195.2 μg/ml, which are three to four orders of magnitude greater than the 95th percentile of exposure in maternal plasma reported in the LIFECODES pregnancy cohort (Bommarito et al., 2021) and only 1.5–2 orders of magnitude greater than the 90th percentile of exposure in serum of pregnant women in the highly exposed C8 Health Study (Stein et al., 2009). In a highly exposed community, human serum concentrations at the 95th percentile of legacy PFAS exposure ranged from 0.001 to 0.028 μg/ml, which is also within two to four orders of magnitude of the concentrations tested in the present study, though serum levels of emerging PFAS such as GenX were not measurable in this population likely due to the timing of discharge control relative to serum sample collection (Kotlarz et al., 2020). Additionally, concentrations tested here overlap with or are within one to two orders of magnitude of those previously shown to induce adverse placental histopathology and fetal growth outcomes in CD-1 mice (1 mg/kg PFOA dosing solution = 241.5 µM, 2 mg/kg GenX dosing solution = 576.2 µM).

During placental development, careful coordination between trophoblast proliferation and apoptosis is critical for a healthy and fully functioning placenta (Huppertz and Herrler, 2005; Pollheimer and Knöfler, 2005), and when disruption of that balance occurs, preeclampsia and intrauterine growth restriction may result (Huppertz and Herrler, 2005). Previous work in JEG-3 trophoblasts has shown PFAS-induced increased expression of pro-apoptotic genes (e.g., *BAD* and *BAX*) and decreased expression of anti-apoptotic genes (e.g., *BCL2* Bangma et al., 2020a). Here we demonstrate the potential for numerous PFAS to disrupt the normal proliferative behavior of placental trophoblasts. It is likely that PFAS disrupt placental development and function through multiple biologic pathways, as we saw both increased and decreased proliferation. A study using the HTR8/SVneo trophoblast cell line demonstrated that exposure to PFOA disrupted the formation of vascular-like structures and activated the NOTCH-pathway, suggesting a potential mechanism of disrupted angiogenesis (Poteser et al., 2020).

Placentae from preeclamptic pregnancies exhibit excessive cell death (Allaire et al., 2000). Here we report significant decreases in trophoblast viability after exposure to PFAS, suggesting this cell type may be involved in the pathogenesis of preeclampsia in exposed populations (Wikström et al., 2019; Bommarito et al., 2021). We also observed significant increases in trophoblast viability, however the viability assay used in this multiplexed HTTS measured a luminescent signal proportional to the amount of ATP released into media after cell lysis, which assumes decreasing levels of ATP correspond to a decreasing number of live cells. The significant increases in cell viability may be the result of cellular release of ATP in response to stress, damage, or necrosis (Bours et al., 2006). Thus, it is possible that the reported increases in cell viability are actually due to stress-related release of ATP by trophoblast cells after PFAS exposure. Previous work has provided evidence linking excess ATP levels to aberrant placentation in preeclampsia (Spaans et al., 2014; Fodor et al., 2020). Further studies are needed to elucidate relationships between the varied mechanisms of PFAS toxicity in trophoblasts.

Trophoblast invasion and migration into the maternal decidua are critical for implantation (Burrows et al., 1996). During this process, trophoblast cells are involved in the remodeling of the maternal spiral arteries, converting them into high capacity, low resistance vessels that ensure an adequate blood supply to the feto-placental unit (Pijnenborg et al., 2006; Hayes et al., 2014). Failure of trophoblast migration and invasion in vascular remodeling are associated with preeclampsia and intrauterine fetal growth restriction (Khong et al., 1986). Our data suggest the potential for certain PFAS to disrupt the normal migration and invasion processes of placental trophoblasts and is consistent with inhibition of trophoblast migration and invasion by PFBS, PFOA, PFOS, and GenX in HTR-8/SVneo cells (Marinello et al. 2020 Szilagyi et al., 2020; Li et al., 2021). Furthermore, we observed gene expression changes in GenX-exposed JEG-3 cells that are consistent with inhibition of trophoblast migration, such as reduced expression of *IGF2* (Irving and Lala, 1995), reduced expression of *EGFR* (Massimiani et al., 2015), and increased expression of *CTNNB1* (Tucker and Nowak, 2018)*.* Future studies will further examine the effects of PFAS on trophoblast migration, particularly at doses lower than those tested here, and potentially using other trophoblast cell models that may be more appropriate for assessing trophoblast invasion, such as HTR-8/SVneo cells, in order to understand the role of PFAS in vascular disorders of pregnancy such as preeclampsia.

Placental mitochondria play a critical role in maintaining a healthy pregnancy, and placental mitochondrial dysfunction occurs in pregnancy conditions such as preeclampsia and gestational diabetes (Fisher et al., 2020). It has been hypothesized that mitochondrial-based signaling (via reactive oxygen species generation) may be the driver of placental adaptations to mitigate damage, thus causing adverse placental outcomes when mitochondria are no longer able to maintain the appropriate reactive oxygen species/anti-oxidant balance (Fisher et al., 2020). Here we provide evidence suggesting PFAS have the potential to disrupt mitochondrial function in trophoblasts. Future investigations should further interrogate the effects of PFAS on trophoblast mitochondrial respiration and reactive oxygen species generation.

Optimal placental and fetal growth is carefully balanced between complex and overlapping systems across the maternal-placental-fetal interface, including the management of inflammation, oxidative stress, endocrine signaling, and the metabolism and transport of nutrients and xenobiotics. After exposure to PFOA or GenX, JEG-3 cells exhibited shifts in gene expression suggesting potential dysregulation of key genes involved in maintaining these processes. ATP Binding Cassette subfamily G Member 2, ABCG2 (also known as breast cancer resistance protein, or BCRP), is localized to the apical membrane of placental syncytiotrophoblasts and functions as an efflux transporter (Mao, 2008). ABCG2 protects the fetus by limiting the transplacental transfer of xenobiotics through transport back into maternal circulation (Mao, 2008). Here we show upregulation of *ABCG2* expression in JEG-3 cells after exposure to GenX (3 or 300 µM) or PFOA (100 µM) for 24 h. Previous work has shown *ABCG2* expression is regulated in placental cells *in vitro* by progesterone and 17β-estradiol (Wang et al., 2006). It is possible that PFOA and GenX influence *ABCG2* expression via noncanonical progesterone receptor signaling pathways (Wang et al., 2006), or that *ABCG2* upregulation is a compensatory response to increase placental efflux of PFOA and GenX in an attempt to limit fetal exposure. Our prior work has shown that between mid- and late gestation in mice, both PFOA and GenX accumulate in gestationally exposed embryos with concentrations increasing over time (Blake et al., 2020).

17β-Hydroxysteroid dehydrogenase 1 (17β-HSD1) serves a dual function in estrogen activation and androgen inactivation and plays a critical role in establishing estradiol (E2) concentration. 17β-HSD1 primarily catalyzes the reduction of estrone (E1) into E2, which is more active. E2-mediated signaling in trophoblasts is hypothesized to play a role in *ABCG2* expression and function (Chatuphonprasert et al., 2018). Previous work has implicated dysregulation of *17β-HSD1* expression in preeclampsia (Ishibashi et al., 2012); however, this study showed decreased expression of *17β-HSD1*. Here we show a significant increase in *17β-HSD1* expression after 24 h of exposure to 100 µM PFOA or 300 µM GenX, which may be the result of ERα induction (Oda et al., 2013) via increased E2 production, but this hypothesis requires further investigation. These divergent findings might also be explained by the post-translational targeting of *17β-HSD1* transcripts by miRNAs upregulated in preeclamptic placentas. It is possible that the post-translational miRNAs involved in regulation of *17β-HSD1* transcripts and downstream protein expression are not fully conserved in JEG-3 cells. Indeed, miRNA expression profiles differ between JEG-3 cells and primary human placental trophoblasts (Morales-Prieto et al., 2012).

Placental oxidative stress is well documented to be strongly associated with the pathogenesis of preeclampsia (Burton and Jauniaux, 2004; Bilodeau, 2014). Glutathione peroxidase 1 (GPX1) protects cells against oxidative damage through catalyzing the reduction of organic hydroperoxides and hydrogen peroxide by glutathione. Furthermore, decreased expression of *GPX1* has been reported in preeclamptic placentas (Vanderlelie et al., 2005; Mistry et al., 2010; Roland-Zejly et al., 2011). Here we show a significant reduction in *GPX1* expression in JEG-3 cells after 24 h of exposure to 3 µM or 300 µM GenX, with a similar non-significant trend of decreased expression after exposure to 100 µM PFOA. In JEG-3 cells exposed to 300 µM GenX, expression of G Protein-Coupled Estrogen Receptor 1 (*GPER1*) and superoxide dismutase 1 (*SOD1*) was disrupted. Previous work has implicated a role for GPER1 in the modulation of oxidative damage in the placenta via inhibition of aldehyde oxidase 1 (*AOX1*), resulting in oxidative damage and impaired function (Maiti et al., 2017). In rats, increased *SOD1* expression has been shown to indicate placental response to oxidative stress, suggesting the increase in *SOD1* expression observed in this study may indicate a compensatory mechanism towards the effects of GenX exposure. Furthermore, exposure to PFOA, PFOS, or GenX in HTR-8/SVneo trophoblasts disrupted the expression of numerous inflammation-related genes (Szilagyi et al., 2020). Taken together, these data suggest PFAS such as GenX may induce gene expression patterns consistent with excess oxidative stress and inflammation, which is associated with adverse pregnancy outcomes like preeclampsia.

Our prior work has shown that both PFOA and GenX similarly disrupt placental weight and histopathology in gestationally-exposed mice, but with some chemical-specific features (Blake et al., 2020). For example, only GenX-exposed placentas had increased thyroxine levels and the incidence of labyrinth atrophy was greater in GenX-exposed placentas while the incidence of labyrinth congestion was greater in PFOA-exposed placentas. The current work provides evidence that could shed light on the similarities and differences between how exposure to PFOA or GenX effects placental trophoblasts, as the gene expression data indicated certain differentially expressed genes were common among both chemicals (e.g., *ABCG2*, *17β-HSD1*) while others were chemical-specific (e.g., *GPER1, MRP3, WNT4*). Although more data are needed, these initial lines of evidence suggest that there are some shared features of toxicity but other toxicological features are likely chemical-specific.

There are several important caveats to this study. First, the concentration-response modeling estimates should not be overinterpreted. This efficient, high-throughput toxicity screen was designed to provide concentration-response model estimates to help inform concentration selection in future experiments. The tradeoff between robustness and efficiency (in terms of both time and cost) is applicable to these data and should be taken into consideration. Second, the multiplexed HTTS design is susceptible to bias due to overt cytotoxicity. While the quality control and data processing implemented in this study attempted to avoid cytotoxicity-driven artifacts, the current experimental workflow precludes the ability to completely prevent such artifacts. Thus, careful attention should be paid to interpreting proliferation and MMP estimates generated in this study, as there is a possibility these parameters were driven by overt cytotoxicity for certain PFAS. Third, the quality control applied on the front end of the migration assay (i.e., starting cellular confluence needed to be ≥70%) resulted in lower biological replicates for certain PFAS, which could reduce precision and potentially introduce bias. And fourth, while the JEG-3 cell line is a useful model of human placental trophoblasts, it is possible that toxicity profiles and gene expression changes may not translate to human biology. These findings would be significantly strengthened by follow up studies utilizing primary trophoblasts or *ex vivo* tissue to confirm the findings reported here.

Lastly, it is possible that external concentration (i.e., the concentration of PFAS in the cell culture media) does not correspond to the bioavailable or internal concentration in JEG-3 cells or other *in vitro* systems. Previous work has shown that PFAS have varying binding affinity towards serum proteins (Jones et al., 2003; Beesoon and Martin, 2015). Given that serum proteins are a necessary and common component of cell culture media, it is possible that the bioavailability of PFAS to the JEG-3 cells is much lower than the concentration in the media. Therefore, it is possible that EC50 estimates reported here underestimate the effects of PFAS towards cell viability, proliferation, and MMP. A recent report showed the intracellular concentration (internal concentration) of PFOA and PFOS was higher after exposure in serum-free media compared to serum-containing media in JEG-3 cells (Bangma et al., 2020a). Similar caution should be taken while interpreting the data from the migration assay, as PFAS bind to serum proteins including those in cell culture media (Bangma et al., 2020a). Thus, the 1-h serum-free pulse may have caused a left shift in the cytotoxicity concentration-response of JEG-3 cells due to increased bioavailability of PFAS.

In this study we developed a high-throughput toxicity screen to evaluate the effect of 42 unique PFAS on placental trophoblast viability and function, explored the impact of six PFAS on trophoblast migration, and examined gene expression changes elicited by exposure to two PFAS selected due to their high public health relevance. Our data provide basic PFAS toxicity data in JEG-3 placental trophoblasts and highlight the potential of these compounds to disrupt critical aspects of trophoblast biology, such as cellular proliferation, migration, and mitochondrial function. We also demonstrate the ability of PFOA and GenX after 24 h of exposure at concentrations as low as 1 and 3 μM, respectively, to induce significant dysregulated expression of placental genes that reside in pathways critical to proper development and function. Taken together, these data suggest placental trophoblasts are a sensitive target of PFAS and further studies to determine mechanisms of toxicity are needed.

## Data Availability

The data presented in the study are deposited in the NTP data repository and are accessible at: https://doi.org/10.22427/NTP-DATA-025-00001-0001-000-9.
