# Supplementary material for: A High-Throughput Toxicity Screen of 42 Per- and Polyfluoroalkyl Substances (PFAS) and Functional Assessment of Migration and Gene Expression in Human Placental Trophoblast Cells
Source: Front Toxicol. 2022 Apr 25;4:881347. doi: 10.3389/ftox.2022.881347 (PMC9081605; doi:10.3389/ftox.2022.881347)
Supplement: Supplementary file 1 [file DataSheet1.docx]

**Supplemental Information**

**A high-throughput toxicity screen of 42 per- and polyfluoroalkyl substances (PFAS) and functional assessment of migration and gene expression in human placental trophoblast cells**

Bevin E. Blake^1,2^, Brittany P. Rickard^1^, and Suzanne E. Fenton^2,*^

^1^Curriculum in Toxicology and Environmental Medicine, University of North Carolina at Chapel Hill, Chapel Hill, NC, USA

^2^Mechanistic Toxicology Branch, Division of the National Toxicology Program, National Institute of Environmental Health Sciences, Research Triangle Park, NC, USA

**Accession link to all data including gene expression data:** https://doi.org/10.22427/NTP-DATA-025-00001-0001-000-9

| **Supplementary Table 1.** List of PFAS tested in the JEG-3 placental cell line, and their unique identifiers, molecular formulas, weights, sources, and purities | | | | | | | |
| --- | --- | --- | --- | --- | --- | --- | --- |
| **Chemical name** | **CASRN** | **Molecular formula** | **Molecular weight** | **DTXSID** | **PubChem CID** | **Manufacturer/Source** | **Purity** |
| Perfluoro-2-methyl-3-oxahexanoic acid | 13252-13-6 | C6HF11O3 | 330.05 | [DTXSID70880215](https://comptox.epa.gov/dashboard/dsstoxdb/results?search=DTXSID70880215) | 114481 | SynQuest Laboratories | 97.0% |
| Perfluoro-3,6-dioxadecanoic acid | 137780-69-9 | C8HF15O4 | 446.07 | [DTXSID50381073](https://comptox.epa.gov/dashboard/dsstoxdb/results?search=DTXSID50381073) | 2778677 | SynQuest Laboratories | 97.0% |
| 8-H-Perfluorooctanoic acid | 13973-14-3 | C8H2F14O2 | 396.08 | [DTXSID70565479](https://comptox.epa.gov/dashboard/dsstoxdb/results?search=DTXSID70565479) | 14922999 | SynQuest Laboratories | 97.0% |
| Perfluoro-3,6-dioxaheptanoic acid | 151772-58-6 | C5HF9O4 | 296.05 | [DTXSID30382063](https://comptox.epa.gov/dashboard/dsstoxdb/results?search=DTXSID30382063) | 2782393 | SynQuest Laboratories | 98.0% |
| Perfluoro-3,6,9-trioxadecanoic acid | 151772-59-7 | C7HF13O5 | 412.06 | [DTXSID80380837](https://comptox.epa.gov/dashboard/dsstoxdb/results?search=DTXSID80380837) | 2778260 | SynQuest Laboratories | 98.0% |
| 7H-Perfluoroheptanoic acid | 1546-95-8 | C7H2F12O2 | 346.07 | [DTXSID70165670](https://comptox.epa.gov/dashboard/dsstoxdb/results?search=DTXSID70165670) | 15243 | SynQuest Laboratories | 98.0% |
| Perfluorooctanesulfonic acid | 1763-23-1 | C8HF17O3S | 500.13 | [DTXSID3031864](https://comptox.epa.gov/dashboard/dsstoxdb/results?search=DTXSID3031864) | 74483 | Sigma-Aldrich | 98.0% |
| Perfluoroundecanoic acid | 2058-94-8 | C11HF21O2 | 564.09 | [DTXSID8047553](https://comptox.epa.gov/dashboard/dsstoxdb/results?search=DTXSID8047553) | 77222 | Oakwood Products | 96.0% |
| 2,2-Difluoro-2-(trifluoromethoxy)acetate sodium salt | 21837-98-9 | C3F5NaO3 | 202.01 | [DTXSID50904660](https://comptox.epa.gov/dashboard/dsstoxdb/results?search=DTXSID50904660) | 88425486 | SynQuest Laboratories | 99.0% |
| Perfluoropentanoic acid | 2706-90-3 | C5HF9O2 | 264.05 | [DTXSID6062599](https://comptox.epa.gov/dashboard/dsstoxdb/results?search=DTXSID6062599) | 75921 | SynQuest Laboratories | 98.0% |
| 6:2 Fluorotelomer sulfonic acid | 27619-97-2 | C8H5F13O3S | 428.16 | [DTXSID6067331](https://comptox.epa.gov/dashboard/dsstoxdb/results?search=DTXSID6067331) | 119688 | Apollo Scientific | 98.0% |
| 2-(N-Ethylperfluorooctanesulfonamido )acetic acid | 2991-50-6 | C12H8F17NO4S | 585.23 | [DTXSID5062760](https://comptox.epa.gov/dashboard/dsstoxdb/results?search=DTXSID5062760) | 18134 | SynQuest Laboratories | 95.0% |
| Perfluorobutylsulfonamide | 30334-69-1 | C4H2F9NO2S | 299.11 | [DTXSID30880251](https://comptox.epa.gov/dashboard/dsstoxdb/results?search=DTXSID30880251) | 10958205 | SynQuest Laboratories | 99.0% |
| Perfluorohexanoic acid | 307-24-4 | C6HF11O2 | 314.05 | [DTXSID3031862](https://comptox.epa.gov/dashboard/dsstoxdb/results?search=DTXSID3031862) | 67542 | SynQuest Laboratories | 97.0% |
| Perfluorododecanoic acid | 307-55-1 | C12HF23O2 | 614.10 | [DTXSID8031861](https://comptox.epa.gov/dashboard/dsstoxdb/results?search=DTXSID8031861) | 67545 | SynQuest Laboratories | 96.0% |
| Perfluoro-3,6,9-trioxatridecanoic acid | 330562-41-9 | C10HF19O5 | 562.08 | [DTXSID50375114](https://comptox.epa.gov/dashboard/dsstoxdb/results?search=DTXSID50375114) | 2760333 | Toronto Research Chemicals | 99.0% |
| Perfluorooctanoic acid | 335-67-1 | C8HF15O2 | 414.07 | [DTXSID8031865](https://comptox.epa.gov/dashboard/dsstoxdb/results?search=DTXSID8031865) | 9554 | Sigma-Aldrich | 99.0% |
| Perfluorodecanoic acid | 335-76-2 | C10HF19O2 | 514.09 | [DTXSID3031860](https://comptox.epa.gov/dashboard/dsstoxdb/results?search=DTXSID3031860) | 9555 | Sigma-Aldrich | 98.0% |
| Perfluorohexanesulfonic acid | 355-46-4 | C6HF13O3S | 400.11 | [DTXSID7040150](https://comptox.epa.gov/dashboard/dsstoxdb/results?search=DTXSID7040150) | 67734 | Sigma-Aldrich | 98.0% |
| Perfluorobutanoic acid | 375-22-4 | C4HF7O2 | 214.04 | [DTXSID4059916](https://comptox.epa.gov/dashboard/dsstoxdb/results?search=DTXSID4059916) | 9777 | SynQuest Laboratories | 98.0% |
| Perfluorobutanesulfonic acid | 375-73-5 | C4HF9O3S | 300.09 | [DTXSID5030030](https://comptox.epa.gov/dashboard/dsstoxdb/results?search=DTXSID5030030) | 67815 | Sigma-Aldrich | 99.6% |
| Perfluoroheptanoic acid | 375-85-9 | C7HF13O2 | 364.06 | [DTXSID1037303](https://comptox.epa.gov/dashboard/dsstoxdb/results?search=DTXSID1037303) | 67818 | Apollo Scientific | 99.0% |
| Perfluoroheptanesulfonic acid | 375-92-8 | C7HF15O3S | 450.12 | [DTXSID8059920](https://comptox.epa.gov/dashboard/dsstoxdb/results?search=DTXSID8059920) | 67820 | SynQuest Laboratories | 95.0% |
| Perfluorononanoic acid | 375-95-1 | C9HF17O2 | 464.08 | [DTXSID8031863](https://comptox.epa.gov/dashboard/dsstoxdb/results?search=DTXSID8031863) | 67821 | Oakwood Products | 100.0% |
| 5H-Octafluoropentanoic acid | 376-72-7 | C5H2F8O2 | 246.06 | [DTXSID50191038](https://comptox.epa.gov/dashboard/dsstoxdb/results?search=DTXSID50191038) | 120227 | SynQuest Laboratories | 97.0% |
| Perfluoro-3-methoxypropanoic acid | 377-73-1 | C4HF7O3 | 230.04 | [DTXSID70191136](https://comptox.epa.gov/dashboard/dsstoxdb/results?search=DTXSID70191136) | 120228 | SynQuest Laboratories | 98.0% |
| 8:2 Fluorotelomer sulfonic acid | 39108-34-4 | C10H5F17O3S | 528.18 | [DTXSID00192353](https://comptox.epa.gov/dashboard/dsstoxdb/results?search=DTXSID00192353) | 3016044 | SynQuest Laboratories | 99.0% |
| Perfluorooctanamide | 423-54-1 | C8H2F15NO | 413.09 | [DTXSID60195123](https://comptox.epa.gov/dashboard/dsstoxdb/results?search=DTXSID60195123) | 67919 | SynQuest Laboratories | 99.0% |
| 6:2 Fluorotelomer phosphate diester | 57677-95-9 | C16H9F26O4P | 790.18 | [DTXSID50561590](https://comptox.epa.gov/dashboard/dsstoxdb/results?search=DTXSID50561590) | 14550408 | Toronto Research Chemicals | 96.0% |
| 6:2 Fluorotelomer phosphate monoester | 57678-01-0 | C8H6F13O4P | 444.09 | [DTXSID90558000](https://comptox.epa.gov/dashboard/dsstoxdb/results?search=DTXSID90558000) | 14250578 | Santa Cruz Biotechnology | 98.0% |
| 8:2 Fluorotelomer phosphate monoester | 57678-03-2 | C10H6F17O4P | 544.10 | [DTXSID60874027](https://comptox.epa.gov/dashboard/dsstoxdb/results?search=DTXSID60874027) | 57351110 | Toronto Research Chemicals | 98.0% |
| Ammonium perfluoro-2-methyl-3-oxahexanoate | 62037-80-3 | C6H4F11NO3 | 347.08 | [DTXSID40108559](https://comptox.epa.gov/dashboard/dsstoxdb/results?search=DTXSID40108559) | 51342034 | SynQuest Laboratories | 98.0% |
| Sodium perfluoropentanesulfonate | 630402-22-1 | C5F11NaO3S | 372.08 | [DTXSID50893449](https://comptox.epa.gov/dashboard/dsstoxdb/results?search=DTXSID50893449) | 87842193 | Toronto Research Chemicals | 95.0% |
| 6:2 Fluorotelomer alcohol | 647-42-7 | C8H5F13O | 364.11 | [DTXSID5044572](https://comptox.epa.gov/dashboard/dsstoxdb/results?search=DTXSID5044572) | 69537 | Sigma-Aldrich | 96.5% |
| 8:2 Fluorotelomer alcohol | 678-39-7 | C10H5F17O | 464.12 | [DTXSID7029904](https://comptox.epa.gov/dashboard/dsstoxdb/results?search=DTXSID7029904) | 69619 | Sigma-Aldrich | 97.0% |
| 8:2 Fluorotelomer phosphate diester | 678-41-1 | C20H9F34O4P | 990.21 | [DTXSID90218051](https://comptox.epa.gov/dashboard/dsstoxdb/results?search=DTXSID90218051) | 3022253 | Santa Cruz Biotechnology | 94.0% |
| Perfluorotridecanoic acid | 72629-94-8 | C13HF25O2 | 664.11 | [DTXSID90868151](https://comptox.epa.gov/dashboard/dsstoxdb/results?search=DTXSID90868151) | 3018355 | Sigma-Aldrich | 97.0% |
| Perfluorooctanesulfonamide | 754-91-6 | C8H2F17NO2S | 499.14 | [DTXSID3038939](https://comptox.epa.gov/dashboard/dsstoxdb/results?search=DTXSID3038939) | 69785 | SynQuest Laboratories | 95.0% |
| 9-H-Perfluorononanoic acid | 76-21-1 | C9H2F16O2 | 446.09 | [DTXSID50226894](https://comptox.epa.gov/dashboard/dsstoxdb/results?search=DTXSID50226894) | 6434 | SynQuest Laboratories | 97.0% |
| Perfluoro(4-methoxybutanoic) acid | 863090-89-5 | C5HF9O3 | 280.05 | [DTXSID60500450](https://comptox.epa.gov/dashboard/dsstoxdb/results?search=DTXSID60500450) | 12498036 | SynQuest Laboratories | 99.0% |
| 1H,1H-Nonafluoropentyl p-toluenesulfonate | 883499-79-4 | C8H5F11O2 | 342.11 | [DTXSID50382065](https://comptox.epa.gov/dashboard/dsstoxdb/results?search=DTXSID50382065) | 14632790 | SynQuest Laboratories | 99.0% |
| 2H,2H,3H,3H-Perfluorooctanoic acid | 914637-49-3 | C12H9F9O3S | 404.25 | [DTXSID20874028](https://comptox.epa.gov/dashboard/dsstoxdb/results?search=DTXSID20874028) | 2782396 | SynQuest Laboratories | 97.0% |

| **Supplementary Table 2.** Raw values for concentration-response curve EC50 estimates from JEG-3 cell viability, proliferation, and mitochondrial membrane potential (MMP) assays after exposure to individual PFAS | | | | | |
| --- | --- | --- | --- | --- | --- |
| **Chemical name** | **CASRN** | **Viability**  **EC50 ± SD (µM)** | **Proliferation EC50 ± SD (µM)** | **MMP**  **EC50 ± SD (µM)** |  |
| Perfluoro-2-methyl-3-oxahexanoic acid | 13252-13-6 | -7645.8 ± NaN | 1109.9 ± 266.6 | -441142.4 ± 10 |  |
| Perfluoro-3,6-dioxadecanoic acid | 137780-69-9 | -35.1 ± 225.2 | 282.8 ± 25.7 | 350.1 ± 16.1 |  |
| 8-H-Perfluorooctanoic acid | 13973-14-3 | 317.5 ± 182.9 | 441.3 ± 18.1 | 147.3 ± 27.1 |  |
| Perfluoro-3,6-dioxaheptanoic acid | 151772-58-6 | 326.5 ± 85.7 | 351.3 ± 11.8 | 368.1 ± 22.6 |  |
| Perfluoro-3,6,9-trioxadecanoic acid | 151772-59-7 | 267.2 ± 26.8 | 302.9 ± 30.1 | 310.1 ± NaN |  |
| 7H-Perfluoroheptanoic acid | 1546-95-8 | 222.8 ± 60.2 | 294.4 ± 48.2 | -254.1 ± 10 |  |
| Perfluorooctanesulfonic acid | 1763-23-1 | 291.2 ± 9.1 | 294.2 ± 5.8 | 352.9 ± 6.7 |  |
| Perfluoroundecanoic acid | 2058-94-8 | 266.1 ± 12.4 | 229.5 ± 13.3 | 200.1 ± 4 |  |
| 2,2-Difluoro-2-(trifluoromethoxy)acetate sodium salt | 21837-98-9 | 2432 ± NA | -38.4 ± NaN | 144436.8 ± 10 |  |
| Perfluoropentanoic acid | 2706-90-3 | 882.2 ± NaN | 831 ± NaN | 174.7 ± 68.4 |  |
| 6:2 Fluorotelomer sulfonic acid | 27619-97-2 | -345.4 ± NaN | 737.1 ± NaN | 70608.3 ± 10 |  |
| 2-(N-Ethylperfluorooctanesulfonamido)acetic acid | 2991-50-6 | 202.7 ± 44.7 | 203.6 ± 17.7 | 100201.9 ± 10 |  |
| Perfluorobutylsulfonamide | 30334-69-1 | -533.2 ± 34.2 | -722.6 ± 104.8 | -495.8 ± 23.2 |  |
| Perfluorohexanoic acid | 307-24-4 | -419.2 ± NaN | 10418.5 ± 10 | 142.4 ± 12.6 |  |
| Perfluorododecanoic acid | 307-55-1 | 361.1 ± 30.8 | 317.8 ± 24.5 | -158 ± NaN |  |
| Perfluoro-3,6,9-trioxatridecanoic acid | 330562-41-9 | 194.9 ± 54.6 | 332.4 ± 34.1 | 29117.5 ± NA |  |
| Perfluorooctanoic acid | 335-67-1 | 357.7 ± 16.9 | 344.1 ± 12.2 | 360.2 ± 18 |  |
| Perfluorodecanoic acid | 335-76-2 | 180.7 ± 25.6 | 234 ± 22.8 | 235.8 ± 159.6 |  |
| Perfluorohexanesulfonic acid | 355-46-4 | 288.7 ± 29.6 | 251.6 ± 21.7 | 86.6 ± 71.5 |  |
| Perfluorobutanoic acid | 375-22-4 | 226 ± 115.7 | 209.2 ± 359.8 | 52 ± 296.5 |  |
| Perfluorobutanesulfonic acid | 375-73-5 | 330.4 ± 35 | 223.2 ± 76.7 | -181.7 ± NaN |  |
| Perfluoroheptanoic acid | 375-85-9 | 486.7 ± 408.6 | 277.4 ± 81.6 | -319.9 ± NaN |  |
| Perfluoroheptanesulfonic acid | 375-92-8 | 1977 ± 2479.7 | 296.2 ± 201.3 | 874.5 ± 1752.1 |  |
| Perfluorononanoic acid | 375-95-1 | 332.6 ± 10.3 | 326.1 ± 5.5 | 304.6 ± 13.6 |  |
| 5H-Octafluoropentanoic acid | 376-72-7 | 353.7 ± 28.6 | 333.7 ± 56.5 | -680 ± 89.5 |  |
| Perfluoro-3-methoxypropanoic acid | 377-73-1 | 303.7 ± 28.2 | 204.1 ± 110.9 | 93.1 ± 131.6 |  |
| 8:2 Fluorotelomer sulfonic acid | 39108-34-4 | 159.5 ± 45.7 | 321.3 ± 171.2 | 331.1 ± 5.7 |  |
| Perfluorooctanamide | 423-54-1 | -261.2 ± NaN | 114 ± 35.6 | 294.6 ± 66.1 |  |
| 6:2 Fluorotelomer phosphate diester | 57677-95-9 | -81.9 ± 638.9 | 141.8 ± 39.2 | 64401.7 ± 10 |  |
| 6:2 Fluorotelomer phosphate monoester | 57678-01-0 | 182.7 ± 21.7 | 193.3 ± 3.8 | 248 ± 62.1 |  |
| 8:2 Fluorotelomer phosphate monoester | 57678-03-2 | 359.8 ± 30.3 | 250.7 ± 37.6 | 312.5 ± 29.8 |  |
| Menadione | 58-27-5 | 205.5 ± 47.1 | 157 ± 21.4 | 195540.8 ± 10 |  |
| Ammonium perfluoro-2-methyl-3-oxahexanoate | 62037-80-3 | -1440 ± 4280.5 | 996.9 ± 952.5 | -120970.2 ± 10 |  |
| Sodium perfluoropentanesulfonate | 630402-22-1 | 1209.2 ± 11424.2 | -1685.5 ± NA | 543.3 ± NaN |  |
| 6:2 Fluorotelomer alcohol | 647-42-7 | 415.4 ± 70.6 | 537.2 ± NaN | 332.4 ± 44.1 |  |
| 8:2 Fluorotelomer alcohol | 678-39-7 | 35.7 ± 36.5 | 19.1 ± NaN | 95.1 ± 15.3 |  |
| 8:2 Fluorotelomer phosphate diester | 678-41-1 | -723.2 ± 131.8 | -167.5 ± NaN | -496.4 ± 816.2 |  |
| Perfluorotridecanoic acid | 72629-94-8 | -111 ± NaN | 388.1 ± 124.9 | -1832.4 ± 10 |  |
| Perfluorooctanesulfonamide | 754-91-6 | 175.9 ± 4.9 | 160 ± 87.8 | 227.4 ± 369.9 |  |
| 9-H-Perfluorononanoic acid | 76-21-1 | 349.9 ± 43.1 | 1227.3 ± 117.5 | 186.9 ± 27.6 |  |
| Perfluoro(4-methoxybutanoic) acid | 863090-89-5 | 17.7 ± NaN | 696.4 ± NaN | -224.5 ± NaN |  |
| 1H,1H-Nonafluoropentyl p-toluenesulfonate | 883499-79-4 | -493.8 ± 41.7 | 306.1 ± 47.9 | 158.6 ± 17.7 |  |
| 2H,2H,3H,3H-Perfluorooctanoic acid | 914637-49-3 | 598.5 ± NaN | 950.6 ± 670.8 | 71779.9 ± 10 |  |

*Abbr*: NaN = not a number, designated as such due to failed model convergence

| **Supplementary Table 3.** Rough binning of PFAS bioactivity in JEG-3 cells using lowest concentration (µM) at which the mean response value exceeded the mean control value ± 2*SD | | | | | | |
| --- | --- | --- | --- | --- | --- | --- |
| **Chemical name** | **CASRN** | **Viability ↓** | **Viability ↑** | **Prolif ↓** | **Prolif ↑** | **MMP ↓** |
| Perfluoro-2-methyl-3-oxahexanoic acid | 13252-13-6 | *na* | *na* | *na* | 450 | 375 |
| Perfluoro-3,6-dioxadecanoic acid | 137780-69-9 | 400 | *na* | 300 | *na* | 400 |
| 8-H-Perfluorooctanoic acid | 13973-14-3 | *na* | *na* | *na* | *na* | *na* |
| Perfluoro-3,6-dioxaheptanoic acid | 151772-58-6 | *na* | 200 | 400 | *na* | 400 |
| Perfluoro-3,6,9-trioxadecanoic acid | 151772-59-7 | *na* | *na* | 350 | *na* | 450 |
| 7H-Perfluoroheptanoic acid | 1546-95-8 | *na* | 50 | 400 | *na* | *na* |
| Perfluorooctanesulfonic acid | 1763-23-1 | 300 | *na* | 250 | *na* | 350 |
| Perfluoroundecanoic acid | 2058-94-8 | 300 | *na* | 100 | *na* | 100 |
| 2,2-Difluoro-2-(trifluoromethoxy)acetate sodium | 21837-98-9 | *na* | *na* | *na* | 50 | *na* |
| Perfluoropentanoic acid | 2706-90-3 | *na* | 200 | *na* | *na* | *na* |
| 6:2 Fluorotelomer sulfonic acid | 27619-97-2 | *na* | *na* | *na* | 50 | *na* |
| 2-(N-Ethylperfluorooctanesulfonamido)acetic acid | 2991-50-6 | 250 | *na* | 250 | 50 | 150 |
| Perfluorobutylsulfonamide | 30334-69-1 | *na* | *na* | *na* | *na* | *na* |
| Perfluorohexanoic acid | 307-24-4 | *na* | 300 | 100 | *na* | 100 |
| Perfluorododecanoic acid | 307-55-1 | *na* | 50 | *na* | *na* | *na* |
| Perfluoro-3,6,9-trioxatridecanoic acid | 330562-41-9 | 300 | *na* | 400 | *na* | 50 |
| Perfluorooctanoic acid | 335-67-1 | *na* | 200 | 350 | *na* | 450 |
| Perfluorodecanoic acid | 335-76-2 | 200 | 50 | 250 | *na* | 50 |
| Perfluorohexanesulfonic acid | 355-46-4 | *na* | 50 | 350 | *na* | *na* |
| Perfluorobutanoic acid | 375-22-4 | *na* | 50 | *na* | *na* | 50 |
| Perfluorobutanesulfonic acid | 375-73-5 | *na* | 50 | *na* | *na* | 50 |
| Perfluoroheptanoic acid | 375-85-9 | *na* | *na* | 450 | *na* | *na* |
| Perfluoroheptanesulfonic acid | 375-92-8 | *na* | *na* | 232.5 | 23.25 | *na* |
| Perfluorononanoic acid | 375-95-1 | 350 | *na* | 300 | *na* | 300 |
| 5H-Octafluoropentanoic acid | 376-72-7 | *na* | *na* | *na* | *na* | *na* |
| Perfluoro-3-methoxypropanoic acid | 377-73-1 | *na* | *na* | *na* | *na* | *na* |
| 8:2 Fluorotelomer sulfonic acid | 39108-34-4 | *na* | *na* | *na* | *na* | *na* |
| Perfluorooctanamide | 423-54-1 | *na* | *na* | *na* | *na* | *na* |
| 6:2 Fluorotelomer phosphate diester | 57677-95-9 | *na* | 112.5 | 187.5 | 37.5 | *na* |
| 6:2 Fluorotelomer phosphate monoester | 57678-01-0 | *na* | *na* | *na* | *na* | *na* |
| 8:2 Fluorotelomer phosphate monoester | 57678-03-2 | *na* | *na* | *na* | *na* | *na* |
| Menadione | 58-27-5 | 50 | *na* | 150 | *na* | 300 |
| Ammonium perfluoro-2-methyl-3-oxahexanoate | 62037-80-3 | 350 | *na* | *na* | 50 | *na* |
| Sodium perfluoropentanesulfonate | 630402-22-1 | 350 | *na* | *na* | 200 | *na* |
| 6:2 Fluorotelomer alcohol | 647-42-7 | *na* | *na* | *na* | *na* | *na* |
| 8:2 Fluorotelomer alcohol | 678-39-7 | *na* | *na* | *na* | *na* | *na* |
| 8:2 Fluorotelomer phosphate diester | 678-41-1 | 50 | *na* | 100 | *na* | 350 |
| Perfluorotridecanoic acid | 72629-94-8 | *na* | 150 | 450 | 50 | *na* |
| Perfluorooctanesulfonamide | 754-91-6 | *na* | *na* | *na* | *na* | *na* |
| 9-H-Perfluorononanoic acid | 76-21-1 | *na* | *na* | *na* | *na* | *na* |
| Perfluoro(4-methoxybutanoic) acid | 863090-89-5 | *na* | *na* | *na* | *na* | *na* |
| 1H,1H-Nonafluoropentyl p-toluenesulfonate | 883499-79-4 | *na* | 50 | 400 | *na* | *na* |
| 2H,2H,3H,3H-Perfluorooctanoic acid | 914637-49-3 | *na* | *na* | *na* | 50 | *na* |
| Abbr: *na* = not available |  |  |  |  |  |  |

| **Supplementary Table 4.** Gene expression data expressed as fold change over untreated control from JEG-3 cells after exposure to PFOA or GenX | | | | | | | | |
| --- | --- | --- | --- | --- | --- | --- | --- | --- |
|  | PFOA 1 µM | | PFOA 100 µM | | GenX 3 µM | | GenX 300 µM | |
| Gene | Fold change | P | Fold change | P | Fold change | P | Fold change | P |
| 11BHSD2 | -1.06 | 0.43 | -1.03 | 0.70 | -1.00 | 1.00 | -1.01 | 0.95 |
| 17BHSD1 | -1.02 | 0.59 | 1.17 | 0.01* | -1.11 | 0.12 | 1.23 | 0.01* |
| ABCG2 | -1.07 | 0.39 | 1.18 | 0.07* | 1.18 | 0.03* | 1.26 | 0.01* |
| AHR | -1.09 | 0.31 | 1.08 | 0.36 | 1.04 | 0.42 | -1.04 | 0.42 |
| AHRR | -1.18 | 0.02* | 1.00 | 1.00 | 1.18 | 0.50 | 1.19 | 0.46 |
| CAT | 1.02 | 0.81 | -1.06 | 0.50 | 1.02 | 0.27 | -1.05 | 0.05* |
| CGB5 | 1.00 | 1.00 | -1.15 | 0.41 | 1.08 | 0.53 | 1.11 | 0.40 |
| COX2 | -1.11 | 0.74 | 1.03 | 0.92 | 1.27 | 0.20 | 1.27 | 0.20 |
| CTNNB1 | 1.00 | 1.00 | 1.00 | 1.00 | 1.00 | 1.00 | 1.15 | <0.01* |
| CYP19A1 | -1.05 | 0.42 | 1.00 | 1.00 | -1.03 | 0.78 | -1.29 | 0.05* |
| CYP1A1 | 1.05 | 0.64 | 1.18 | 0.15 | -1.22 | 0.14 | -1.16 | 0.25 |
| CYP2E1 | -1.20 | 0.08 | 1.00 | 1.00 | -1.26 | 0.36 | 1.04 | 0.86 |
| CYP3A5 | 1.28 | 0.30 | -1.18 | 0.46 | 1.19 | 0.60 | -1.83 | 0.10* |
| CYP3A7 | 1.15 | 0.52 | -1.22 | 0.36 | 1.01 | 0.93 | -1.25 | 0.07* |
| EGFR | 1.07 | 0.47 | -1.07 | 0.46 | 1.00 | 1.00 | -1.22 | 0.07* |
| ENG | 1.02 | 0.81 | -1.19 | 0.10* | 1.04 | 0.42 | -1.04 | 0.42 |
| ESRRA | 1.00 | 1.00 | 1.00 | 1.00 | 1.00 | 1.00 | 1.19 | 0.05* |
| ESRRG | -1.04 | 0.86 | -1.16 | 0.47 | -1.16 | 0.21 | 1.00 | 1.00 |
| GH2 | 1.02 | 0.86 | 1.12 | 0.29 | *na* | *na* | *na* | *na* |
| GHR | 1.00 | 1.00 | 1.15 | 0.22 | 1.15 | 0.11 | 1.11 | 0.20 |
| GPER1 | 1.02 | 0.76 | -1.05 | 0.54 | -1.02 | 0.86 | 1.34 | 0.06* |
| GPX1 | 1.00 | 1.00 | -1.14 | 0.27 | -1.18 | 0.07* | -1.29 | 0.01* |
| HIF1A | 1.00 | 1.00 | 1.00 | 1.00 | 1.00 | 1.00 | 1.05 | 0.27 |
| IGF2 | 1.00 | 1.00 | -1.00 | 1.00 | 1.00 | 1.00 | -1.38 | 0.01* |
| IGFBP5 | -1.13 | 0.32 | -1.02 | 0.85 | *na* | *na* | *na* | *na* |
| IGFR1 | 1.03 | 0.75 | 1.08 | 0.38 | -1.02 | 0.77 | -1.10 | 0.24 |
| IGFR2 | 1.05 | 0.46 | 1.16 | 0.04* | 1.04 | 0.56 | -1.00 | 0.95 |
| IL6 | -1.05 | 0.84 | 1.10 | 0.70 | -1.23 | 0.34 | -1.51 | 0.09* |
| MRP1 | -1.03 | 0.60 | -1.06 | 0.32 | -1.05 | 0.57 | 1.14 | 0.20 |
| MRP2 | 1.09 | 0.65 | -1.03 | 0.87 | 1.05 | 0.82 | 1.00 | 1.00 |
| MRP3 | -1.08 | 0.66 | -1.12 | 0.51 | 1.07 | 0.64 | 1.44 | 0.05* |
| mTOR | 1.00 | 1.00 | 1.00 | 1.00 | 1.00 | 1.00 | 1.00 | 1.00 |
| NFKB1 | 1.00 | 1.00 | 1.03 | 0.27 | 1.03 | 0.27 | 1.03 | 0.27 |
| NFKB2 | 1.00 | 1.00 | 1.00 | 1.00 | -1.07 | 0.13 | -1.04 | 0.42 |
| NR3C1 | 1.09 | 0.27 | 1.00 | 1.00 | 1.00 | 1.00 | 1.00 | 1.00 |
| PGF | 1.00 | 1.00 | -1.00 | 1.00 | 1.00 | 1.00 | -1.00 | 1.00 |
| PMM1 | -1.03 | 0.70 | 1.07 | 0.32 | -1.05 | 0.41 | 1.05 | 0.43 |
| ROLYR2L | 1.00 | 1.00 | 1.00 | 1.00 | 1.00 | 1.00 | 1.00 | 1.00 |
| SLC2A1 | -1.02 | 0.88 | -1.11 | 0.39 | -1.04 | 0.48 | -1.13 | 0.04* |
| SOD1 | 1.00 | 1.00 | 1.00 | 1.00 | 1.00 | 1.00 | 1.14 | 0.05* |
| TGFB1 | 1.00 | 1.00 | -1.18 | 0.16 | -1.04 | 0.51 | 1.00 | 1.00 |
| VEGFA | 1.00 | 1.00 | 1.08 | 0.11 | 1.04 | 0.37 | 1.27 | <0.01* |
| VEGFB | -1.00 | 0.95 | -1.04 | 0.36 | 1.00 | 1.00 | -1.01 | 0.87 |
| WNT4 | 1.25 | 0.26 | 1.45 | 0.09* | 1.08 | 0.64 | 1.20 | 0.26 |
| WNT6 | -1.04 | 0.84 | -1.02 | 0.91 | -1.09 | 0.43 | -1.09 | 0.43 |
| WNT7B | 1.06 | 0.39 | 1.06 | 0.39 | 1.07 | 0.55 | -1.12 | 0.36 |

*Abbr:* na = not available due to insufficient probe hybridization

**
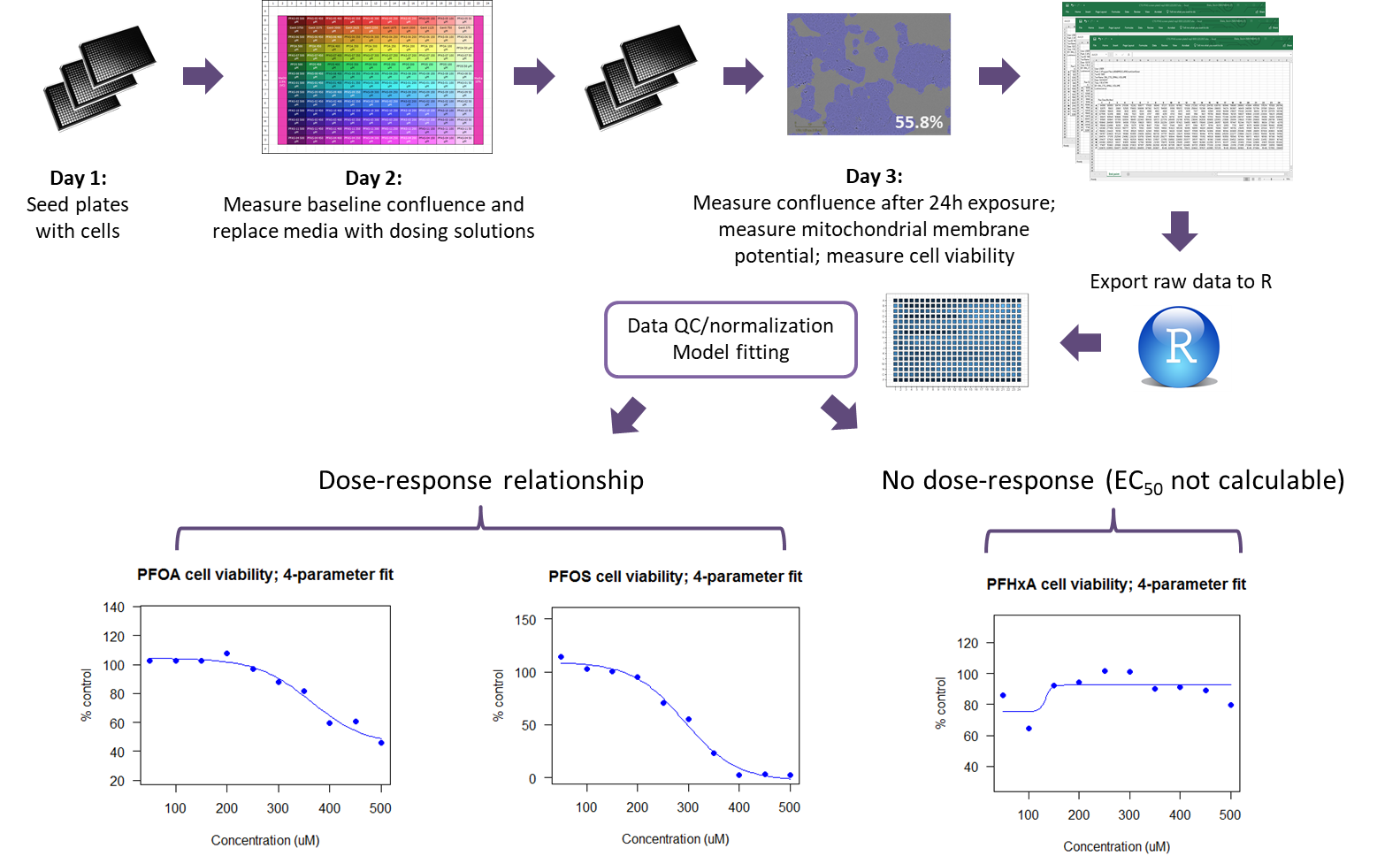
**

**Supplementary Figure 1.** Experimental workflow to conduct efficient high-throughput toxicity screening of PFAS using JEG-3 cells. JEG-3 cells were seeded in 384 well plates and exposed to test chemicals using a liquid handling device. Test chemicals were randomized and experimenters were blinded to their identity during the exposure period. Cell growth was monitored over the 24-hour exposure period using a live cell imager. Mitochondrial membrane potential and cell viability assays were multiplexed and read by fluorescence and luminescence intensities, respectively. Raw data were imported into R and processed using a custom script. Four parameter dose-response curves were fit to the data and model estimates were extracted.


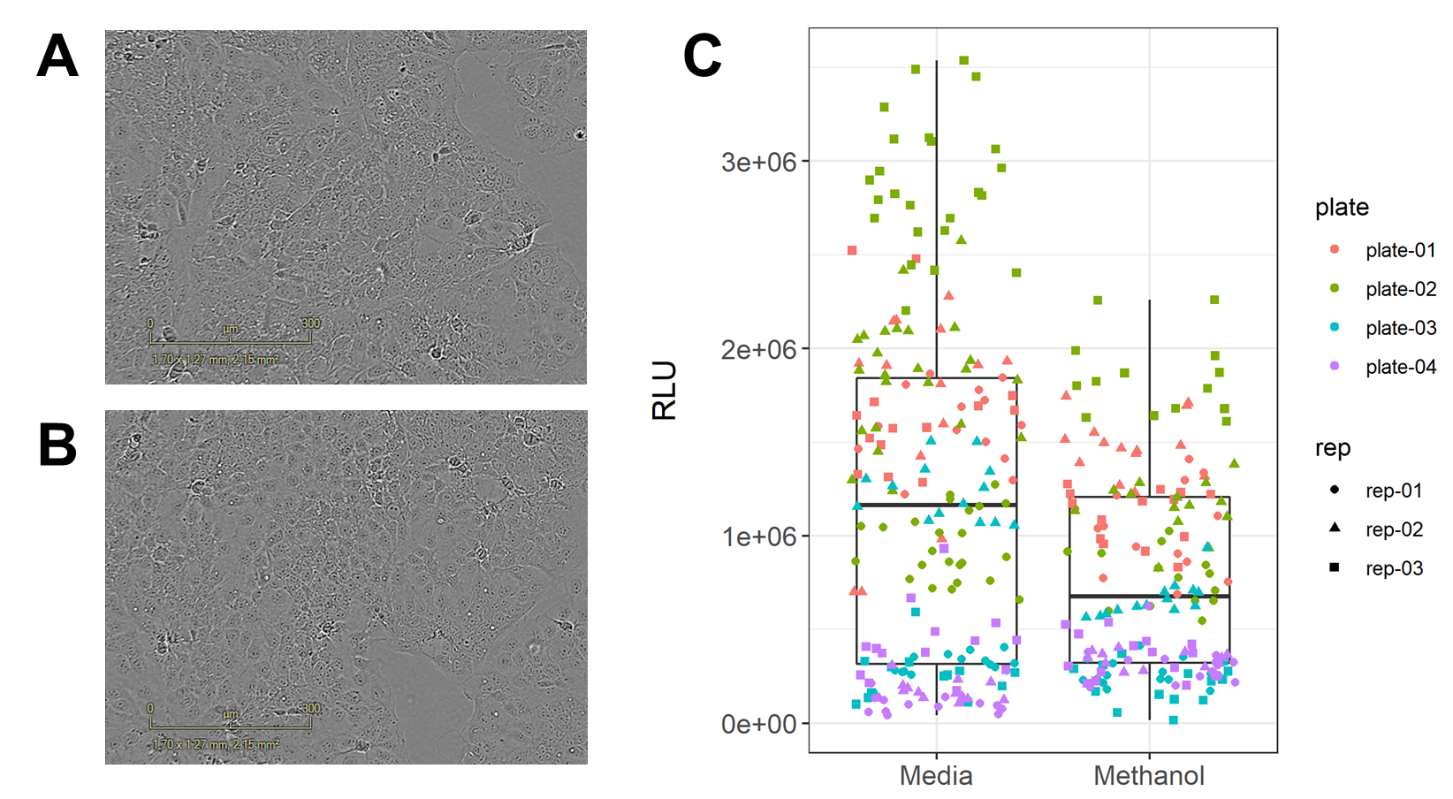


**Supplementary Figure 2.** Representative phase contrast live cell images and raw viability relative luminescence units (RLU) in JEG-3 cells after 24 hours exposure to media only or vehicle control (2% methanol, 98% media). (A) Representative image of JEG-3 cells exposed to media only. (B) Representative image of JEG-3 cells exposed to vehicle control. (C) Boxplots and raw cell viability RLU values for experimental replicates (N = 3, denoted by point shape) for experimental plates 1-4 (plate denoted by point color). The boxplot upper and lower hinges correspond to the first and third quartiles (25th and 75th percentiles), the middle hinge corresponds to the median, and the upper whisker extends to the highest value that is within 1.5 times the distance between the first and third quartiles (inter-quartile range, IQR) of the hinge and the lower whisker extends to the lowest value within 1.5 times the IQR of the hinge.


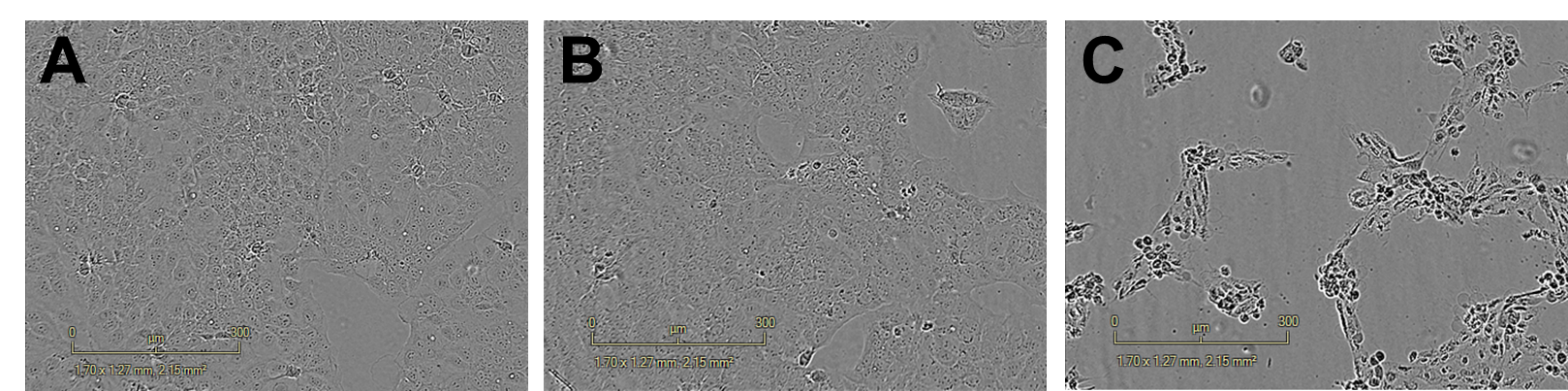


**Supplementary Figure 3.** Representative images of JEG-3 cells after 24 hours exposure to (A) 0 µM, (B) 100 µM, and (C) 150 µM menadione (positive control for cell death). Complete cell death is apparent after exposure to 150 µM, while minimal cell death is visible at 100 µM.


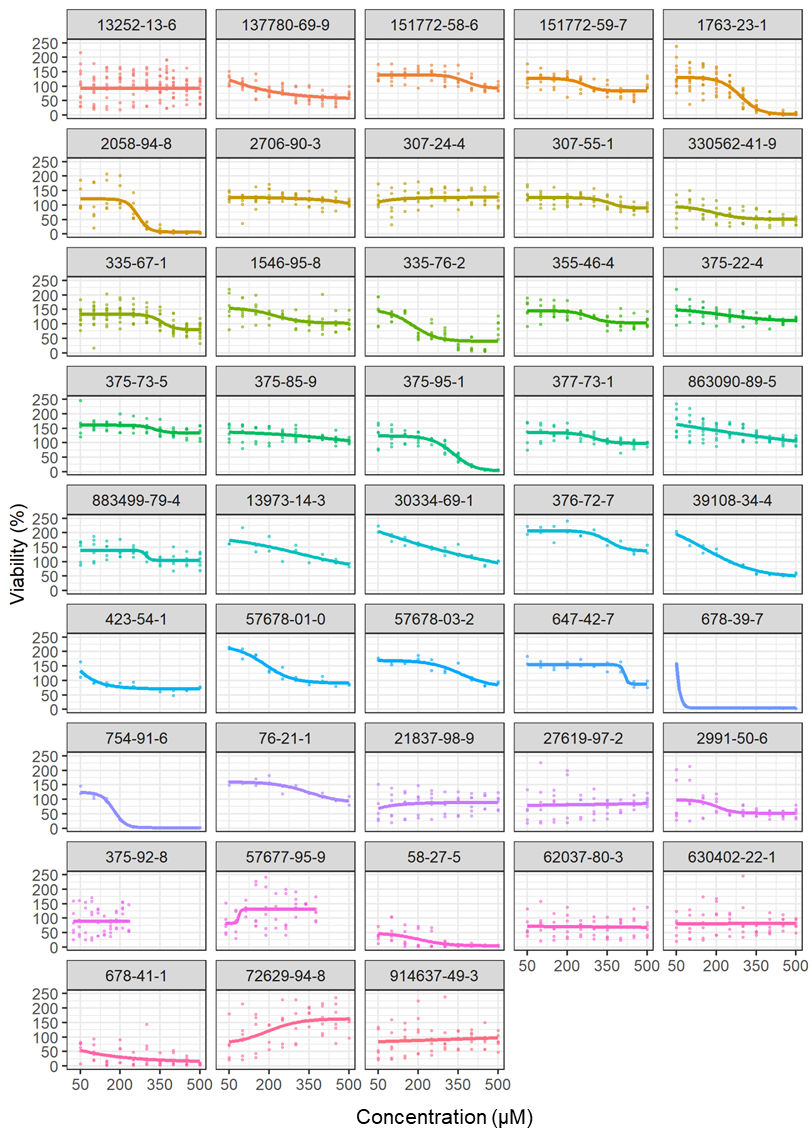


**Supplementary Figure 4.** Concentration-response model fits for cell viability after 24 hours exposure to PFAS. CAS numbers are shown in the grey header of individual plots. Data were fit to a four-parameter concentration-response model with no constraints and EC50 estimates were extracted. N = 3 biological replicates.


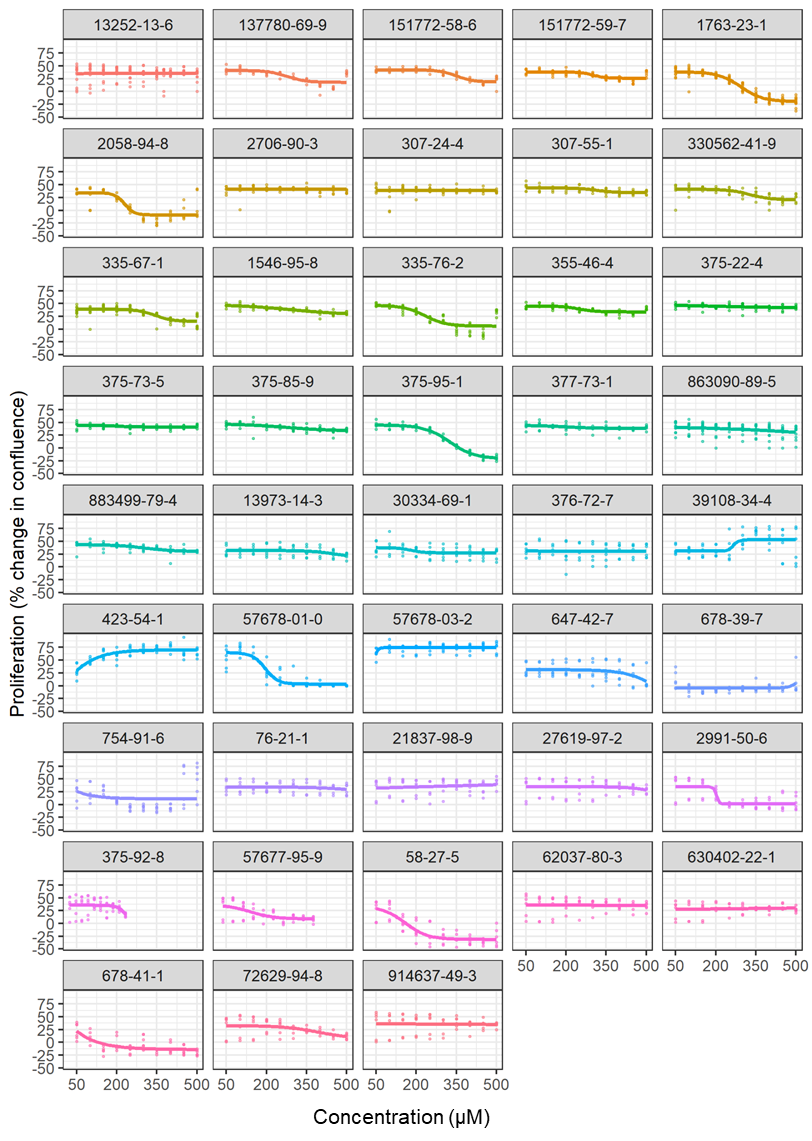


**Supplementary Figure 5.** Concentration-response model fits for cell proliferation after 24 hours exposure to PFAS. CAS numbers are shown in the grey header of individual plots. Data were fit to a four-parameter concentration-response model with no constraints and EC50 estimates were extracted. N = 3 biological replicates.


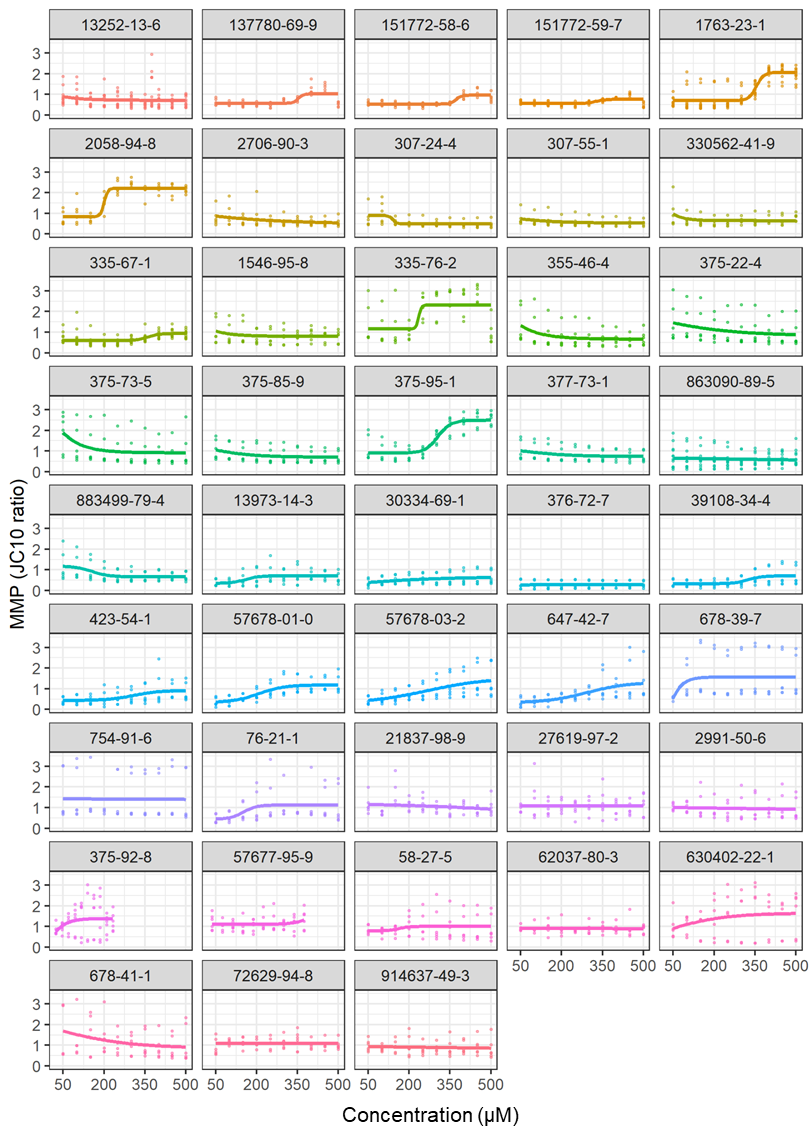


**Supplementary Figure 6.** Concentration-response model fits for cell mitochondrial membrane potential (MMP) after 24 hours exposure to PFAS. CAS numbers are shown in the grey header of individual plots. MMP was determined by the JC-10 ratio assay. Data were fit to a four-parameter concentration-response model with no constraints and EC50 estimates were extracted. N = 3 biological replicates.


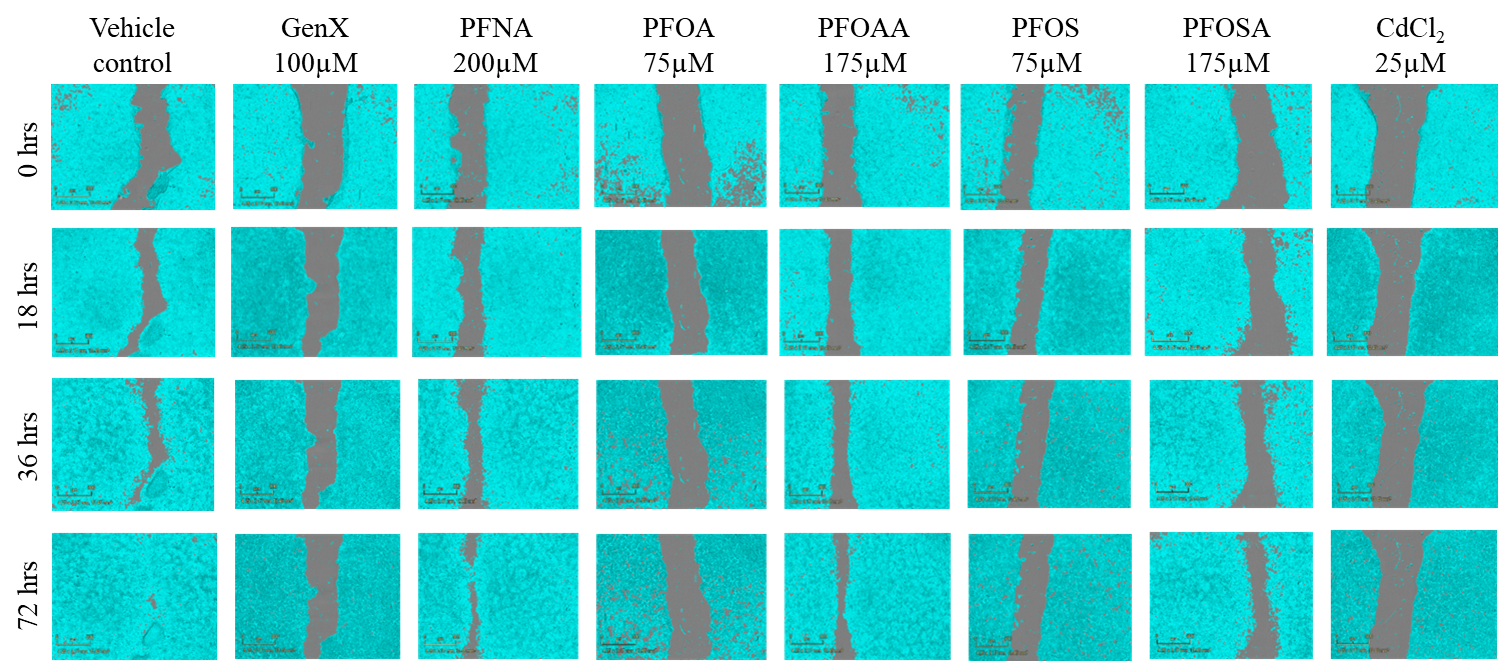


**Supplementary Figure 7.** Effects of PFAS on JEG-3 cell migration during wound healing after 0, 18, 36, and 72 hours of exposure. Each column represents time points of a different PFAS at specific concentration: A) vehicle control, B) 100 µM GenX, C) 200 µM PFNA, D) 75 µM PFOA, E) 175 µM PFOAA, F) 75 µM PFOS, G) 175 µM PFOSA, H) 25 µM cadmium chloride. JEG-3 cells are masked in teal while regions without cells (the wound) are shown in grey.


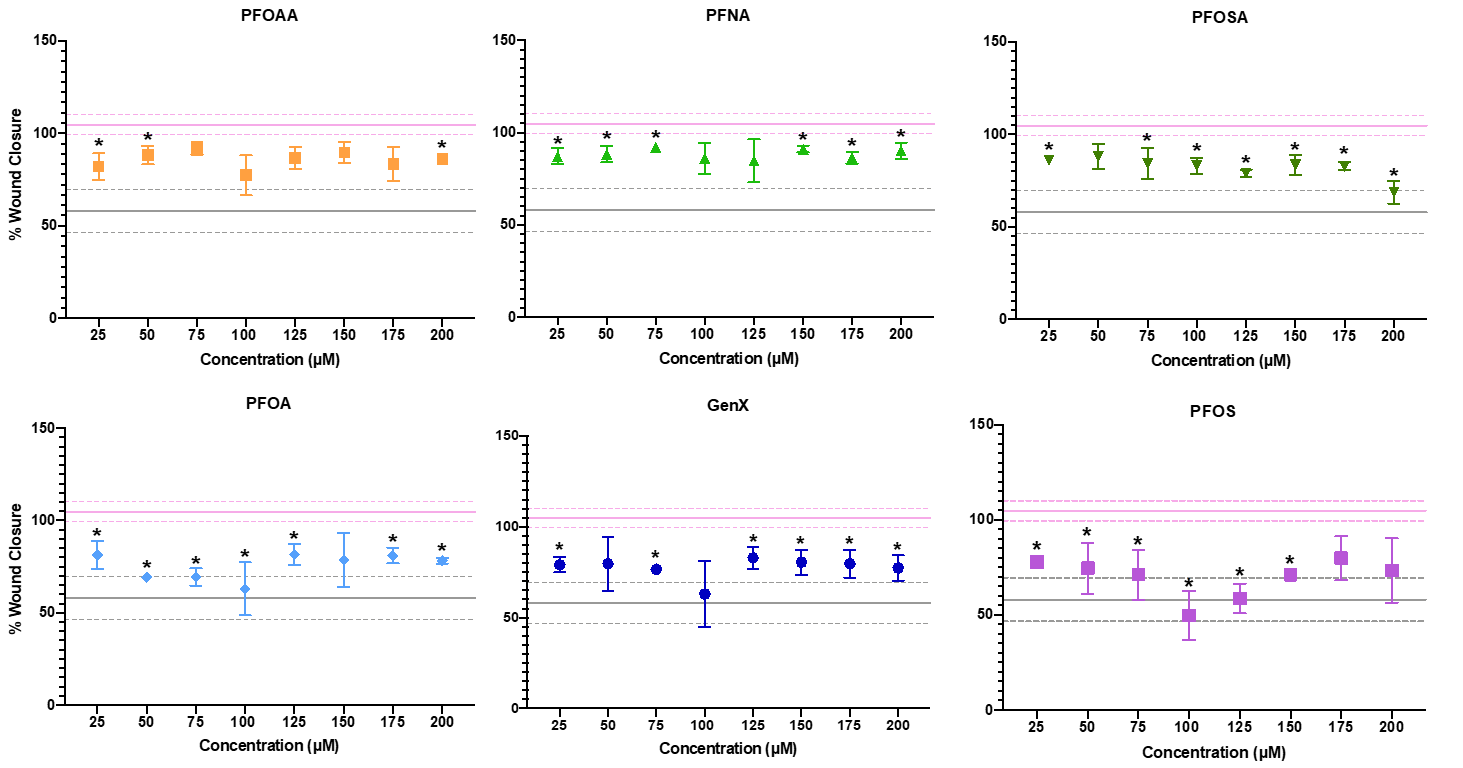


**Supplementary Figure 8.** JEG-3 cell migration after 48 hours of exposure to select PFAS, assessed using a scratch wound assay. For each chemical, migration is expressed as % wound closure, which measures the extent to which JEG-3 cells migrated across the scratch wound between when the initial scratch was made and 48 hours post-scratch (mean ± SD). The upper pink solid line flanked by dotted lines represents the mean ± SD for vehicle control (2% methanol). The lower gray solid line flanked by dotted lines represents the mean ± SD values for the positive control (25 µM cadmium chloride). N = 2-4 biological replicates per chemical and concentration. Significance was determined using multiple unpaired t-tests comparing experimental conditions to the vehicle control followed by *post hoc* correction using the Bonferroni-Dunn method, *p<0.05.


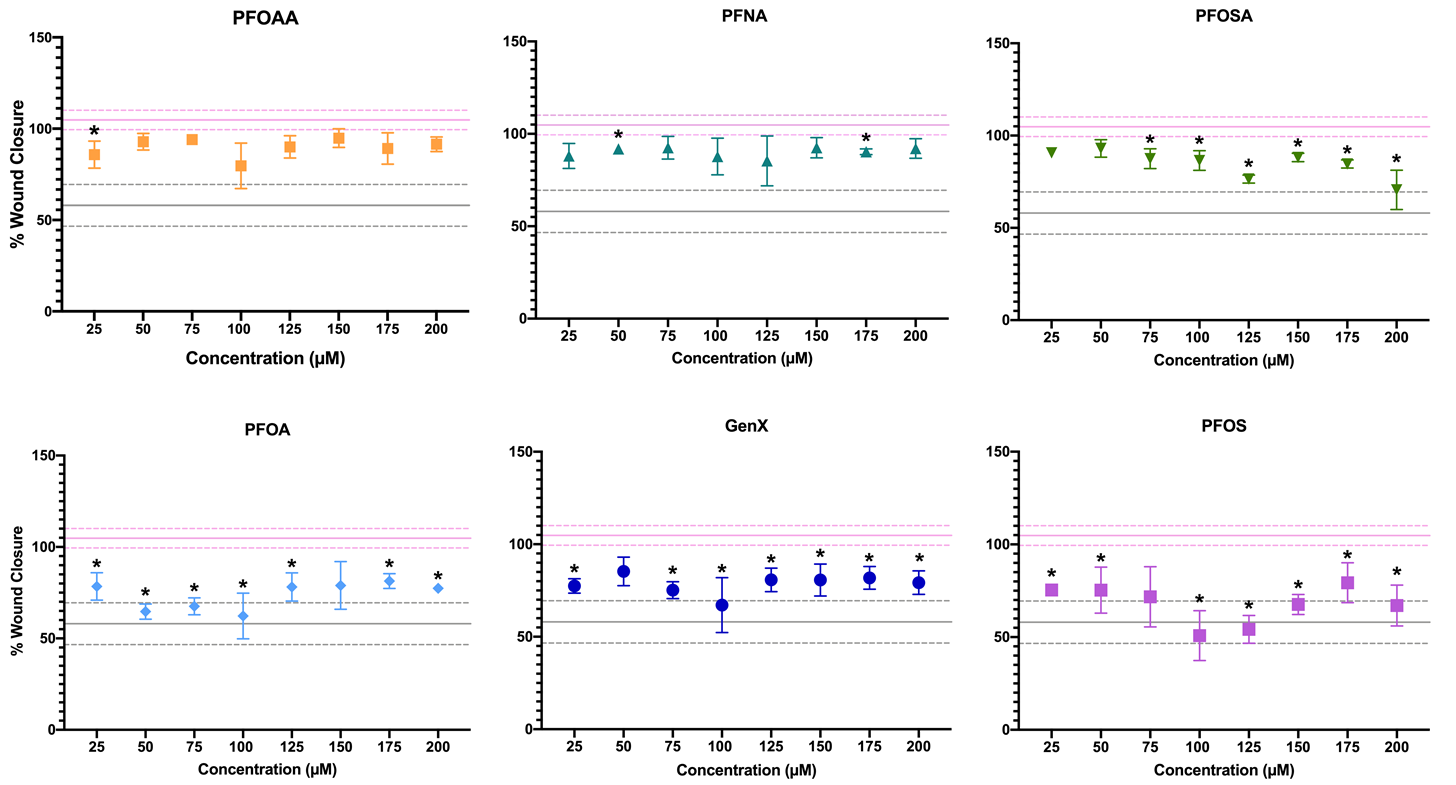


**Supplementary Figure 9.** JEG-3 cell migration after 72 hours of exposure to select PFAS, assessed using a scratch wound assay. For each chemical, migration is expressed as % wound closure, which measures the extent to which JEG-3 cells migrated across the scratch wound between when the initial scratch was made and 72 hours post-scratch (mean ± SD). The upper pink solid line flanked by dotted lines represents the mean ± SD for vehicle control (2% methanol). The lower gray solid line flanked by dotted lines represents the mean ± SD values for the positive control (25 µM cadmium chloride). N = 2-4 biological replicates per chemical and concentration. Significance was determined using multiple unpaired t-tests comparing experimental conditions to the vehicle control followed by *post hoc* correction using the Bonferroni-Dunn method, *p<0.05.
